# Characterisation of a Betasatellite Associated With Tomato Yellow Leaf Curl Guangdong Virus and Discovery of an Unusual Modulation of Virus Infection Associated With C4 Protein

**DOI:** 10.1111/mpp.70051

**Published:** 2025-01-14

**Authors:** Zhenggang Li, Yafei Tang, Xiaoman She, Lin Yu, Guobing Lan, Shanwen Ding, Zifu He

**Affiliations:** ^1^ Guangdong Provincial Key Laboratory of High Technology for Plant Protection, Plant Protection Research Institute Guangdong Academy of Agricultural Sciences Guangzhou China

**Keywords:** betasatellite, C4, DNA methylation, modulation, tomato yellow leaf curl Guangdong virus, virus infection

## Abstract

Tomato yellow leaf curl Guangdong virus (TYLCGdV), a monopartite begomovirus first identified in 2004, remains poorly characterised. In this study, we demonstrate that TYLCGdV associates with a betasatellite, TYLCGdB, and the βC1 protein encoded by TYLCGdB is essential for symptom development. We also explore the role of TYLCGdV C4 protein by generating a C4‐deficient infectious clone (TYLCGdV_mC4_), revealing a dynamic role for TYLCGdV C4. Specifically, viral accumulation in TYLCGdV_mC4_/TYLCGdB‐inoculated plants was significantly lower than that in TYLCGdV/TYLCGdB‐inoculated plants at 7 and 14 days post‐inoculation (dpi), but surpassed that of TYLCGdV/TYLCGdB‐inoculated plants by 25 dpi. Furthermore, although C4 proteins in other begomoviruses typically exhibit one or more of the following properties: (i) suppression of post‐transcriptional gene silencing (PTGS), (ii) suppression of transcriptional gene silencing (TGS), (iii) enhancement of pathogenicity in potato virus X (PVX) and (iv) symptom induction when transgenically expressed, TYLCGdV C4 did not exhibit any of these properties. However, the dynamic role of TYLCGdV C4 in viral infection appears to result from its effects on viral DNA methylation. At 7 dpi, the cytosine methylation level in the TYLCGdVmC4 genome was notably elevated compared to that of the wild‐type virus. However, this trend reversed by 14 dpi, with the wild‐type virus exhibiting a higher methylation level. By 25 dpi, the cytosine methylation levels of both TYLCGdVmC4 and TYLCGdV were comparable. These results indicate that TYLCGdV C4 modulates viral infection via an unconventional mechanism. This novel observation highlights the need for further investigation into the diverse roles of C4 proteins in begomoviruses.

## Graphical abstract

The C4 protein, encoded by tomato yellow leaf curl Guangdong virus (TYLCGdV), does not inhibit PTGS or TGS; the C4 protein modulates TYLCGdV infection by regulating the cytosine methylation level of viral genome at different infection stages.

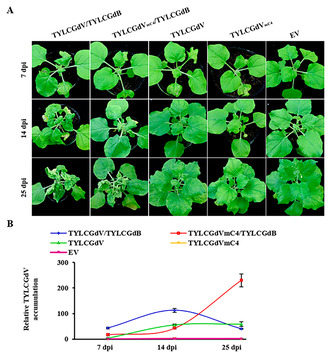

## 1 Introduction

Geminiviruses are among the most devastating viruses, causing severe disease symptoms on vegetables, cereals, and ornamental plants in most tropical and subtropical regions in the world. The genome of geminiviruses is single‐stranded circular DNA with a length of 2.5–5.2 kb (Hanley‐Bowdoin et al. 1999; Fauquet et al. 2008). Geminiviruses are transmitted by different kinds of insect species, depending on different genera. The family *Geminiviridae* contains 14 genera: *Becurtovirus*, *Begomovirus*, *Capulavirus*, *Citlodavirus*, *Curtovirus*, *Eragrovirus*, *Grablovirus*, *Maldovirus*, *Mastrevirus*, *Mulcrilevirus*, *Opunvirus*, *Topilevirus*, *Topocuvirus* and *Turncurtovirus* (Zerbini et al. 2017; Fiallo‐Olive et al. 2021). With the exception of the genus *Begomovirus*, viruses in the other genera have monopartite genomes.

The genus *Begomovirus* consists of 445 species and is the largest genus among plant viruses (Zerbini et al. 2017; Fiallo‐Olive et al. 2021). *Begomovirus* species contain mono‐ or bipartite genomes and infect only dicots. Monopartite begomoviruses contain only one circular single‐stranded DNA (ssDNA), which encodes six proteins: V1 and V2 on the virion‐sense strand, and C1, C2, C3 and C4 on the complementary‐sense strand (Hanley‐Bowdoin et al. 1999). Recent studies have demonstrated that begomoviruses also encode some small proteins that are essential for viral infection, such as C5 and V3 (Zhao et al. 2022; Gong et al. 2021). Bipartite begomoviruses contain two circular ssDNA components, DNA A and DNA B. DNA A is similar to monopartite begomoviruses, whereas DNA B encodes two proteins: BV1 on the virion‐sense strand and BC1 on the complementary‐sense strand.

Satellites are circular ssDNA of about half or a quarter the size of the begomovirus DNA A component. Betasatellites, about half the size of monopartite begomovirus DNA A, are often associated with Old World (OW) monopartite begomoviruses and encode the βC1 protein on the complementary strand. Betasatellites require their helper viruses to replicate and spread. βC1 has been shown to suppress both post‐transcriptional gene silencing (PTGS) and transcriptional gene silencing (TGS), and to act as the pathogenic determinant (Cui et al. 2005, 2004; Yang et al. 2019; Li et al. 2018; Zhou 2013). The betasatellite associated with tomato yellow leaf curl China virus (TYLCCNV) has been shown to encode a novel protein, βV1, on the virion‐sense strand (Hu et al. 2020). βV1 is also a pathogenic determinant and can elicit a strong hypersensitive response (HR)‐type cell death in *Nicotiana benthamiana* leaves (Hu et al. 2020).

C4/AC4, the least conserved protein among geminivirus proteins, plays powerful and diverse roles during viral infection (Medina‐Puche et al. 2021). C4/AC4 is known for acting as a symptom determinant and for suppressing both PTGS and TGS (Rigden et al. 1994; Stanley and Latham 1992; Zhan et al. 2018; Xie et al. 2013; Ismayil et al. 2018; Gopal et al. 2007; Li et al. 2020; Medina‐Puche et al. 2021). Transgenic expression of C4/AC4 can induce abnormal growth of plants, with a phenotype very similar to the symptoms of viral infection (Mei et al. 2018; Mills‐Lujan and Deom 2010; Luna et al. 2012; Rosas‐Diaz et al. 2018). C4 has been shown to be essential for monopartite begomovirus infection, and disruption of C4 expression substantially reduces virus infection (Li, Zeng, et al. 2018; Zhao, Li, et al. 2024). However, the AC4 protein of some bipartite begomoviruses plays no role in virus infection (Elmer et al. 1988; Hong and Stanley 1995; Hoogstraten, Hanson, and Maxwell 1996).

DNA methylation is a crucial epigenetic modification that plays a significant role in regulating gene expression and maintaining genome stability. DNA methylation has been shown to play an important role during geminivirus infection. Previous studies have shown that geminivirus DNA genomes are also methylated in infected plants, and this methylation can attenuate geminivirus virulence (Zarreen and Chakraborty 2020; Wang et al. 2020, 2019). Cytosine methylation substantially reduced the replication of tomato golden mosaic virus (TGMV), demonstrating that DNA methylation can inhibit the replication of geminiviruses (Brough et al. 1992). The tomato resistance genes *Ty‐1*/*Ty‐3* encode RNA‐dependent RNA polymerase RDRγ, which increases cytosine methylation of the geminivirus genome and mediates tomato resistance to TYLCV (Butterbach et al. 2014). Hypermethylation of the intergenic region and AC1 coding region of tomato leaf curl New Delhi virus (ToLCNDV) can endow tomato plants with stronger resistance levels (Sahu et al. 2014). Another study has shown that potato spindle tuber viroid (PSTVd) can increase the expression of tomato DNA methylation‐related genes DRM2, CMT, MET1, AGO4, and HEN1, leading to high methylation of tomato yellow leaf curl Sardinia virus (TYLCSV), reducing the replication and transcription of TYLCSV (Torchetti et al. 2016).

To evade methylation, geminiviruses have developed various strategies, such as encoding viral proteins that interfere with the host methylation cycle, including de novo methylation and methylation maintenance (Guo et al. 2022). Cotton leaf curl Multan virus (CLCuMuV) C4 protein interferes with the host DNA methylation cycle by inhibiting the activity of a core enzyme, S‐adenosyl methionine synthetase (SAMS), that is essential for the methylation process (Ismayil et al. 2018). The βC1 protein encoded by tomato yellow leaf curl China betasatellite (TYLCCNB) interacts with the key protein SAHH in the methylation cycle and inhibits its activity to suppress TGS (Yang, Xie, et al. 2011). In addition, TYLCCNB βC1 also decreases viral DNA methylation and promotes viral virulence by facilitating the DNA glycosylase activity of DEMETER (DME) (Gui et al. 2022).

Tomato yellow leaf curl Guangdong virus (TYLCGdV) was first found in Guangdong province in 2004 and is a monopartite begomovirus (He, Yu, and Luo 2005). Although the DNA A component of TYLCGdV was cloned and analysed, the pathogenicity of TYLCGdV has not been studied. Moreover, the presence of a betasatellite and its role during viral infection remain unclear. Furthermore, the function of TYLCGdV C4 protein is yet to be elucidated. In this study, we found successful infection of TYLCGdV requires a betasatellite, especially the βC1 protein. The C4 protein neither suppressed PTGS nor TGS. TYLCGdV_mC4_, with C4 disruption, still systemically infected *N. benthamiana*, and the virus accumulation was much higher than that of wild‐type (WT) virus at the later stages of infection. Time‐course methylation analysis suggested that C4 may modulate viral virulence by regulating the cytosine methylation level of the TYLCGdV genome. We have discovered an unusual modulation of the C4 protein in regulating begomovirus infection.

## 2 Results

### 2.1 Betasatellite Is Indispensable for TYLCGdV Symptom Development

**FIGURE 1:**
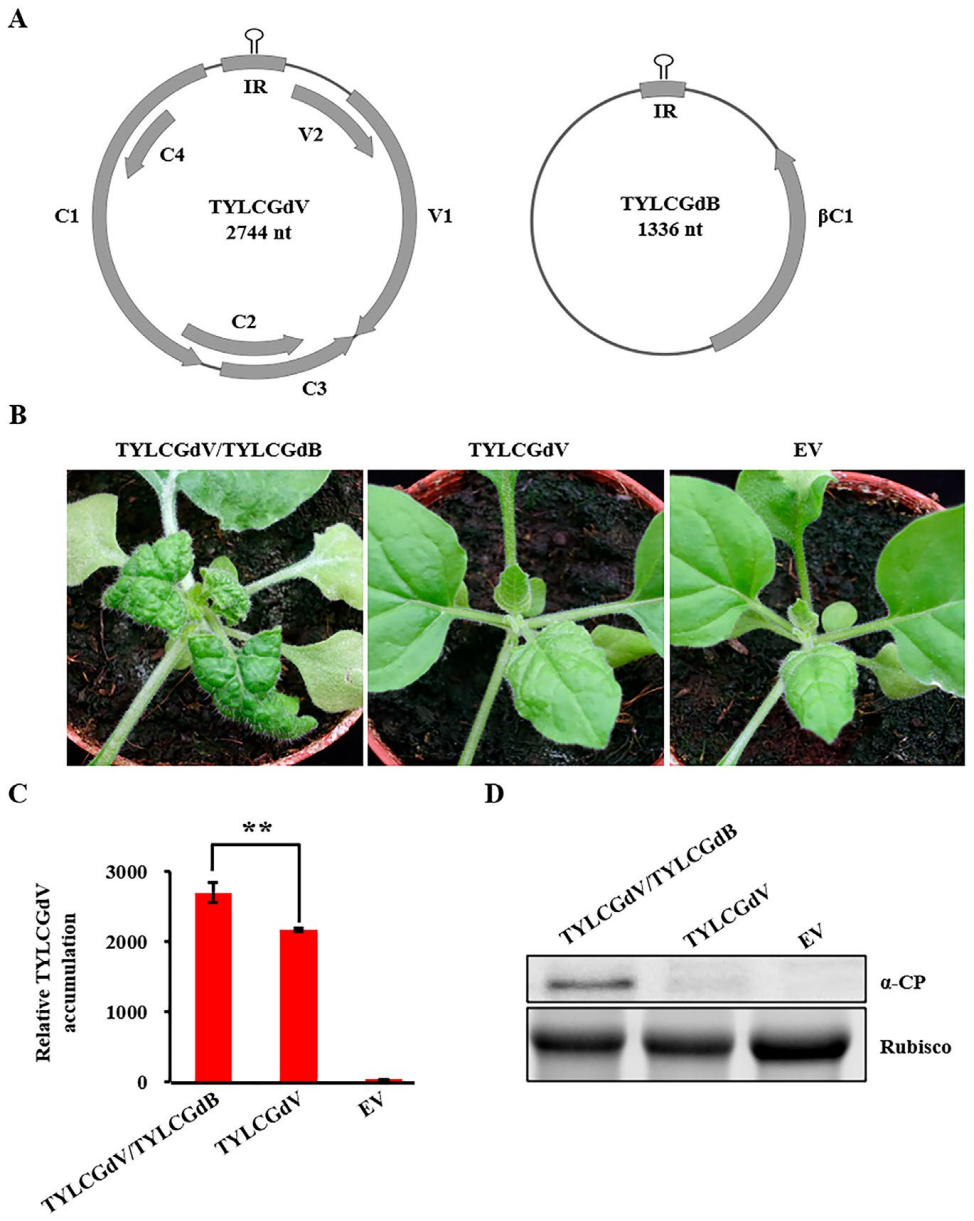
Betasatellite is crucial for TYLCGdV symptom development. (A) Schematic diagram of the genomes of TYLCGdV and betasatellite. The full length of TYLCGdV and TYLCGdB is 2744 nucleotides (nt) and 1336 nt, respectively. TYLCGdV encodes six proteins, and TYLCGdB encodes one protein. IR, intergenic region. (B) Symptoms of *Nicotiana benthamiana* plants inoculated with TYLCGdV with betasatellite (TYLCGdV/TYLCGdB), TYLCGdV alone or empty vector (EV) control at 10 days post‐inoculation. This experiment was repeated three times with six plants in each treatment. (C) Quantitative PCR detection of viral DNA accumulation in plants infected by TYLCGdV/TYLCGdB or TYLCGdV alone. The accumulation values are the average data from three biological replicates. An asterisk indicates the *p* value between TYLCGdV/TYLCGdB and TYLCGdV according to the Student's *t* test (two‐tailed). **p < 0.01. The error bar indicates the *SD*. *NbEF1α* was used as the reference gene. (D) Western blot detection of coat protein (CP) accumulation in plants infected by TYLCGdV/TYLCGdB or TYLCGdV alone with anti‐CP antibody. RuBisCO was used as an equal loading control.

The TYLCGdV sequence comprises 2744 nucleotides (nt) and encodes six proteins, which are designated as V1, V2, C1, C2, C3 and C4 (Figure 1A). From the samples collected in Baiyun district in 2004, we identified that TYLCGdV harbours a betasatellite, designated as TYLCGdB. TYLCGdB is a circular ssDNA comprising 1336 nt and encodes a protein on the complementary‐sense strand, βC1 (Figure 1A).

To study the molecular characteristics of TYLCGdV, we constructed the infectious clones of TYLCGdV and TYLCGdB using primer pairs shown in Table S1. pGreenII‐TYLCGdV contains 1.3‐mer tandem repeat sequence of TYLCGdV, and pGreenII‐TYLCGdB contains a 1.5‐mer tandem repeat sequence of the betasatellite (Figure S1). *Agrobacterium* strains containing pGreenII‐TYLCGdV alone, or the mixed strains harbouring pGreenII‐TYLCGdV and pGreenII‐TYLCGdB at a 1:1 ratio, or the strain carrying empty vector (EV) alone, were infiltrated into 5‐ to 6‐leaf‐stage *N. benthamiana* plants. At 10 days post‐inoculation (dpi), *N. benthamiana* plants infected by TYLCGdV/TYLCGdB showed significant leaf curling and yellowing symptoms (Figure 1B), whereas *N. benthamiana* plants agroinfiltrated with TYLCGdV showed no visible disease symptoms compared with EV (Figure 1B). To our surprise, quantitative PCR (qPCR) detection showed that the virus accumulation of TYLCGdV in the upper leaves was nearly three‐quarters of that of TYLCGdV/TYLCGdB (Figure 1C). However, coat protein (CP) accumulation of TYLCGdV was much lower than that of TYLCGdV/TYLCGdB (Figure 1D). These results demonstrated that although TYLCGdV alone could infect *N. benthamiana*, the betasatellite is indispensable for symptom development and contributes to the viral accumulation.

### 2.2 βC1 Is Required for TYLCGdV Symptom Development

**FIGURE 2:**
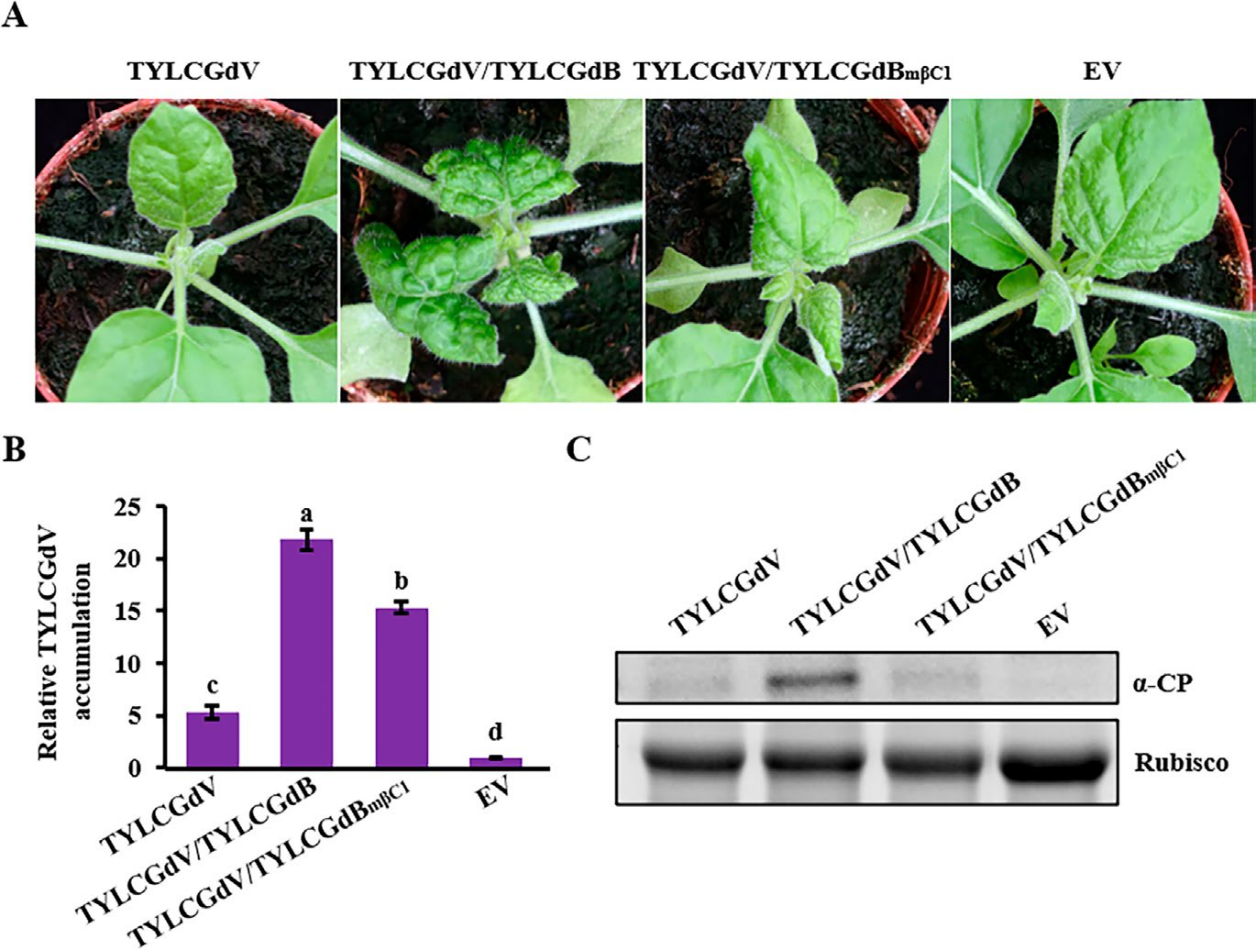
βC1 protein is required for disease symptom development in *Nicotiana benthamiana*. (A) Symptoms of *N. benthamiana* plants inoculated with TYLCGdV alone, TYLCGdV with betasatellite (TYLCGdV/TYLCGdB), a βC1‐null mutant (TYLCGdV/TYLCGdB_mβC1_) or empty vector (EV) control at 10 days post‐inoculation. (B) Viral DNA accumulation of TYLCGdV alone, TYLCGdV with betasatellite (TYLCGdV/TYLCGdB) or a βC1‐null mutant (TYLCGdV/TYLCGdB_mβC1_) in plants by quantitative PCR detection. Different letters indicate significant differences among treatments using Duncan's multiple range test. This experiment was repeated three times with similar results. (C) Western blot detection of coat protein (CP) accumulation in plants infected by TYLCGdV, TYLCGdV/TYLCGdB or TYLCGdV/TYLCGdB_mβC1_ with anti‐CP antibody. RuBisCO was used as an equal loading control.

Betasatellite encodes a single protein, βC1, on the complementary‐sense strand. To investigate the role of βC1 protein during the infection of TYLCGdV, we developed an infectious clone of the betasatellite, designated as pGreenII‐TYLCGdB_mβC1_, which features a targeted disruption in the expression of βC1. pGreenII‐TYLCGdB_mβC1_ also incorporates a 1.5‐mer tandem repeat sequence of betasatellite. Mutations were introduced at the methionine residues located at positions 1, 9 and 16, with the aim of completely abolishing the expression of the βC1 protein. The *Agrobacterium* strain containing the pGreenII‐TYLCGdV plasmid was mixed with the strain carrying either the pGreenII‐TYLCGdB or the pGreenII‐TYLCGdB_mβC1_ plasmid at a 1:1 ratio, followed by co‐infiltration into *N. benthamiana* plants. TYLCGdV and EV were used as individual controls. At 10 dpi, *N. benthamiana* plants infected by TYLCGdV/TYLCGdB_mβC1_ showed much milder symptoms compared with plants infected by TYLCGdV/TYLCGdB (Figure 2A). qPCR and western blot analyses also demonstrated that virus accumulation in plants infected by TYLCGdV/TYLCGdB_mβC1_ was reduced compared to that in plants infected by TYLCGdV/TYLCGdB (Figure 2B,C). These results revealed that βC1 is required for TYLCGdV infection in *N. benthamiana*.

### 2.3 Phylogenetic Analysis of TYLCGdV C4 Protein

**FIGURE 3:**
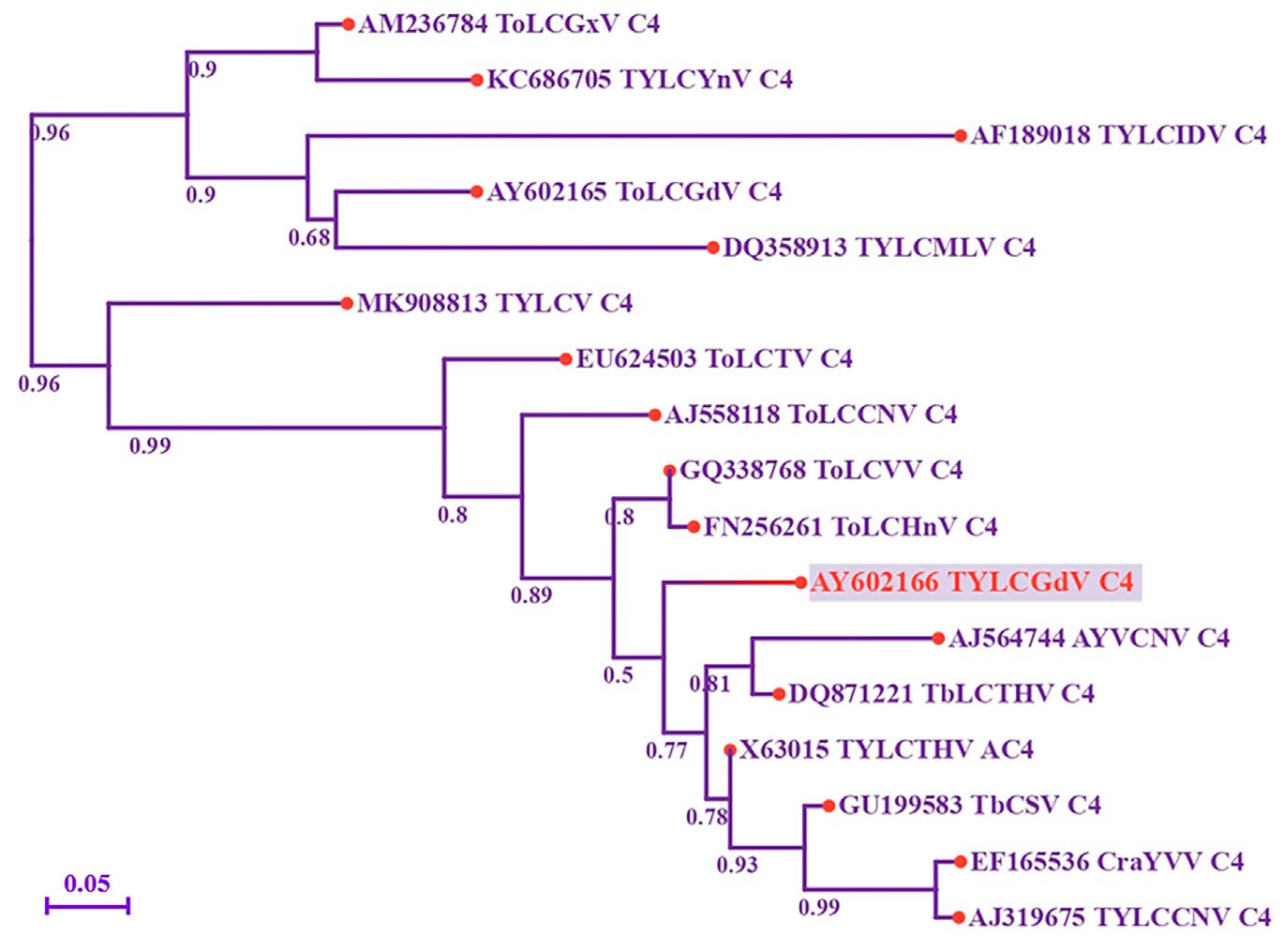
Phylogenetic analysis of C4/AC4 protein sequences was performed using the neighbour‐joining method. Seventeen C4/AC4 protein sequences were obtained from the NCBI database. Information about the begomoviruses used for analysis is listed in Table 1. Numbers on the branches represent bootstrap obtained from 1000 replicates. TYLCGdV C4 is marked in red.

C4/AC4 is one of the most diverse proteins among geminiviruses. To explore the phylogenetic relationship of TYLCGdV C4, a phylogenetic tree was constructed using the protein sequences of begomovirus C4/AC4 (Table 1). The results showed that TYLCGdV C4 shares the highest amino acid identity with the C4 protein of tomato leaf curl Vietnam virus (ToLCVV) (91.75%) and tomato leaf curl Hainan virus (ToLCHnV) (89.69%) (Figure 3). TYLCGdV C4 is also closely related to the C4 protein of tobacco leaf curl Thailand virus (TbLCTHV) (87.63%) and tomato yellow leaf curl China virus (TYLCCNV) (85.57%) (Figure 3). However, TYLCGdV C4 has greater genetic distance from the C4 protein of tomato leaf curl Guangdong virus (ToLCGdV) (42.27%) (Figure S2), tomato yellow leaf curl Yunnan virus (TYLCYnV) (49.48%) and tomato yellow leaf curl virus (TYLCV) (68.04%) (Figure 3).

**TABLE 1:** Begomoviruses used in the phylogenetic analyses.

Abbreviation of virus name	Species	Accession number		Origin
		DNA A	DNA B	
AYVCNV	Ageratum yellow vein China virus	AJ564744		China: Hainan 1.19:2001
CraYVV	Crassocephalum yellow vein virus	EF165536		China: Jinhong:2005
TbCSV	Tobacco curly shoot virus	GU199583		China: Alternanthera:2008
TbLCTHV	Tobacco leaf curl Thailand virus	DQ871221		Thailand: Tomato:2005
ToLCCNV	Tomato leaf curl China virus	AJ558118		China: Guangxi 32:2002
ToLCGdV	Tomato leaf curl Guangdong virus	AY602165		China: Guangzhou 2:2003
ToLCGxV	Tomato leaf curl Guangxi virus	AM236784		China: Guangxi 1:2003
ToLCHnV	Tomato leaf curl Hainan virus	FN256261		China: Hainan:HK7:2008
ToLCTV	Tomato leaf curl Taiwan virus	EU624503		China: Hong Kong T1:2007
ToLCVV	Tomato leaf curl Vietnam virus	GQ338768		Vietnam: Haiduong 122:2008
TYLCCNV	Tomato yellow leaf curl China virus	AJ319675		China: Yunnan 10: Tobacco:2000
TYLCGdV	Tomato yellow leaf curl Guangdong virus	AY602166		China: Guangzhou 3:2003
TYLCIDV	Tomato yellow leaf curl Indonesia virus	AF189018		Indonesia: Lembang:2005
TYLCMLV	Tomato yellow leaf curl Mali virus	DQ358913		Ethiopia: Melkassa:2005
TYLCTHV	Tomato yellow leaf curl Thailand virus	X63015	X63016	Thailand: 1
TYLCV	Tomato yellow leaf curl virus	MK908813		China: Hainan
TYLCYnV	Tomato yellow leaf curl Yunnan virus	KC686705		China: Yunnan

### 2.4 C4 Protein Contributes to Virus Infection at an Early Stage and Negatively Regulates Virus Infection at the Late Stage

**FIGURE 4:**
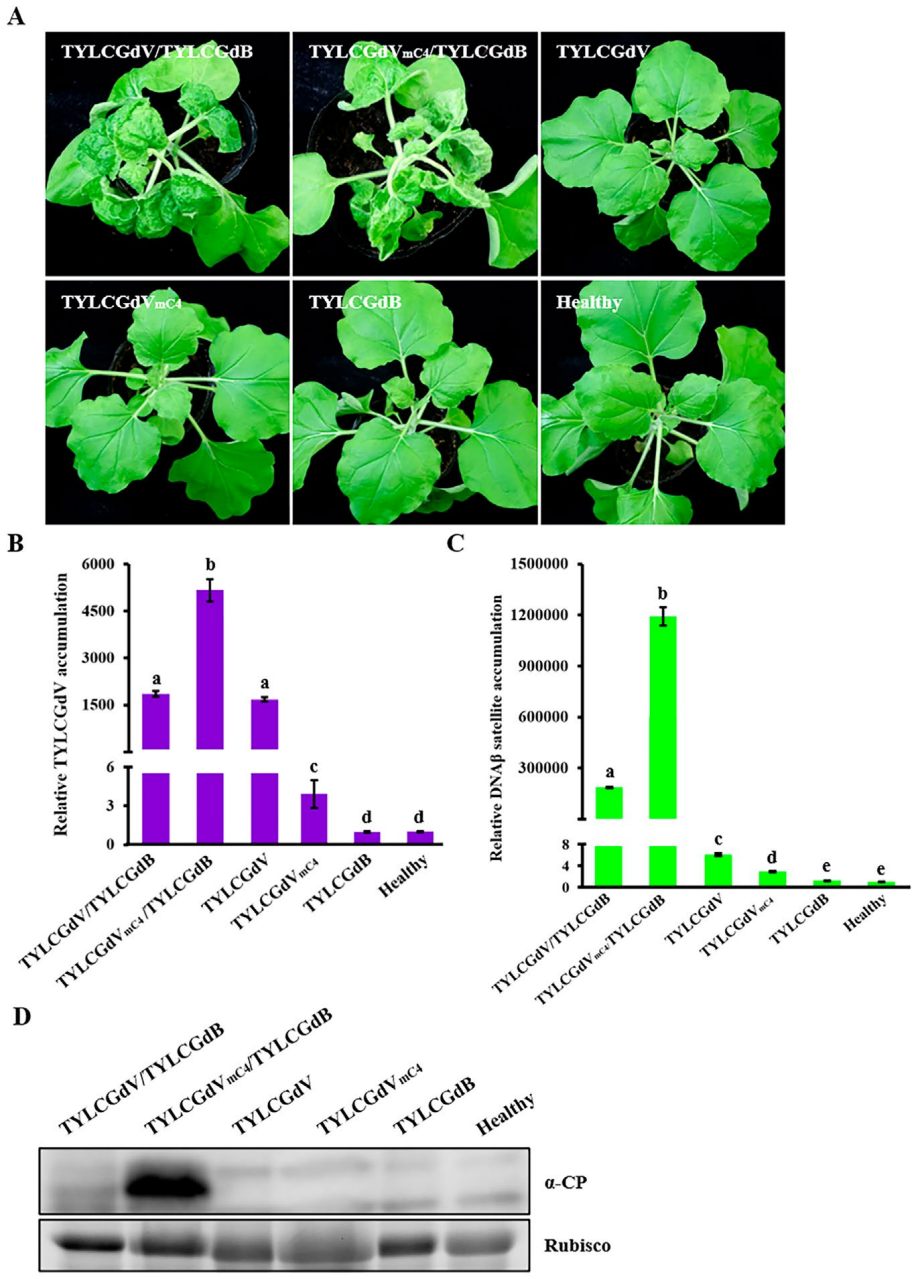
Disruption of C4 does not affect TYLCGdV infection. (A) Symptoms of *Nicotiana benthamiana* plants infected by TYLCGdV with betasatellite (TYLCGdV/TYLCGdB), a C4‐null mutant (TYLCGdV_mC4_/TYLCGdB), TYLCGdV alone, a C4‐null mutant without betasatellite (TYLCGdV_mC4_) or betasatellite alone (TYLCGdB). Photographs were taken at 23 days post‐inoculation. (B, C) Quantitative PCR detection of the viral accumulation of TYLCGdV and betasatellite in the plants from (A). Different letters in the figures indicate significant differences as determined by Duncan's multiple range test. This experiment was repeated three times with similar results, and each treatment included three plants. (D) Western blot detection of coat protein (CP) accumulation in plants from (A) using anti‐CP antibody. RuBisCO was used as an equal loading control.

The function of C4 protein varies between different geminiviruses (Medina‐Puche et al. 2021). To explore the function of C4 protein during TYLCGdV infection, the start codon ATG of the C4 open reading frame (ORF) was replaced by ACG, which has no effect on the expression of Rep protein. *Agrobacterium* strains carrying either the pGreenII‐TYLCGdV or pGreenII‐TYLCGdV_mC4_ plasmid were mixed with a strain containing pGreenII‐TYLCGdB plasmid at a 1:1 ratio, and then co‐infiltrated into *N. benthamiana* plants. At 23 dpi, although all *N. benthamiana* plants infected by TYLCGdV/TYLCGdB or TYLCGdV_mC4_/TYLCGdB showed disease symptoms, including downward leaf curling, crinkling, and mosaic, TYLCGdV_mC4_/TYLCGdB caused more severe symptoms (Figure 4A). To assess the recovery of the C4 start codon mutation, the C4 fragment was amplified and sequenced, and the results showed no recovery mutation (data not shown). qPCR detection also showed that the accumulation of both the virus and the betasatellite in TYLCGdV_mC4_/TYLCGdB‐infected plants were much higher than that in TYLCGdV/TYLCGdB‐infected plants (Figure 4B,C). Western blot detection with anti‐CP antibodies further demonstrated that CP accumulation in plants infected by TYLCGdV_mC4_/TYLCGdB was much higher than that in plants infected by TYLCGdV/TYLCGdB (Figure 4D).

**FIGURE 5:**
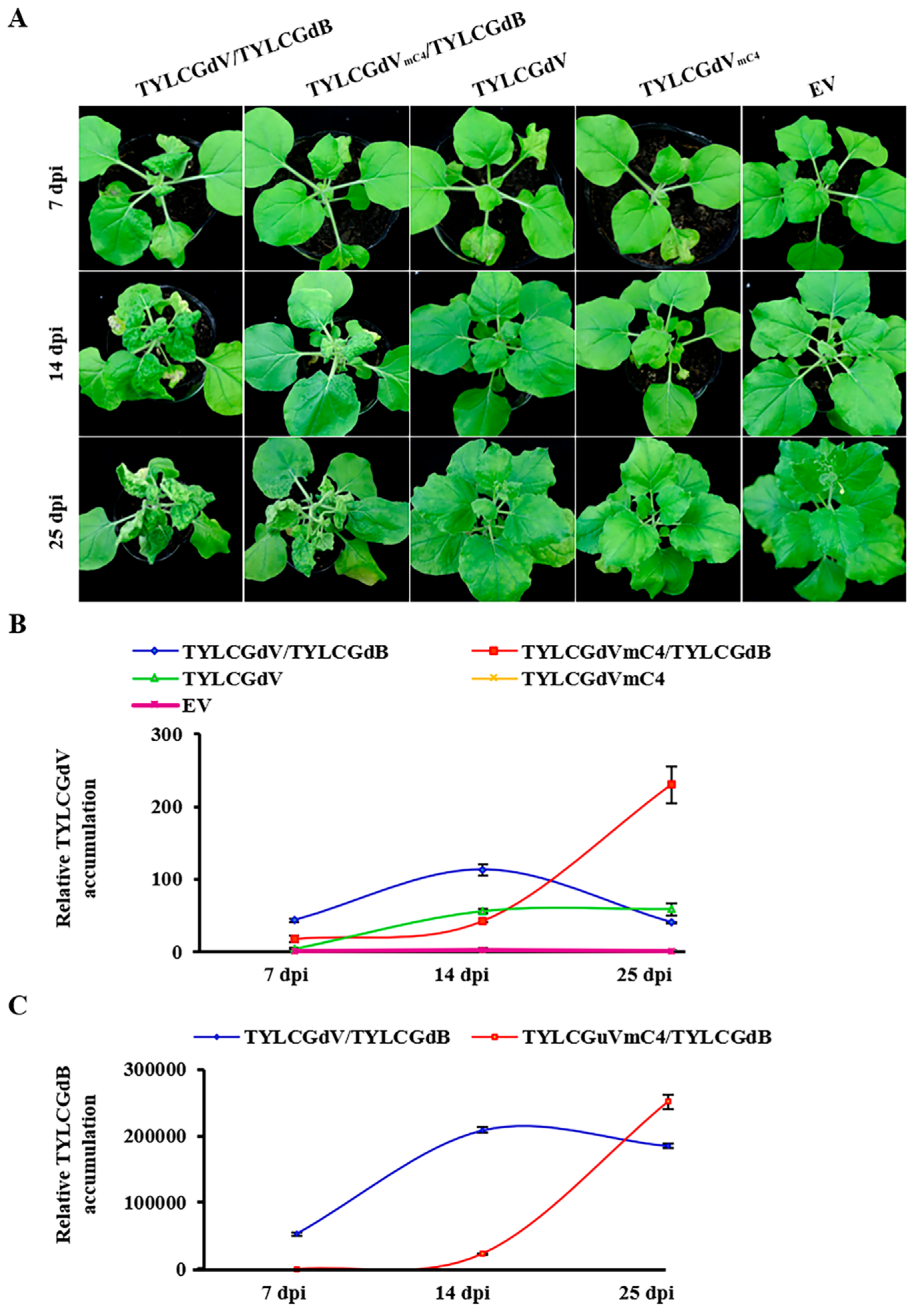
C4 protein negatively regulates the late‐stage infection of TYLCGdV. (A) Symptom development of *Nicotiana benthamiana* plants inoculated with TYLCGdV with betasatellite (TYLCGdV/TYLCGdB), a C4‐null mutant (TYLCGdV_mC4_/TYLCGdB), TYLCGdV alone or a C4‐null mutant without betasatellite (TYLCGdV_mC4_) at 7, 14 and 25 days post‐inoculation (dpi). (B) Quantitative PCR (qPCR) detection of viral DNA accumulation in the plants from (A). Samples were taken at 7, 14 and 25 dpi for qPCR detection. Different colours indicate different treatments. This experiment was repeated three times with the same results. The error bar indicates the *SD*. (C) qPCR detection of betasatellite DNA accumulation in the plants infected by TYLCGdV/TYLCGdB or TYLCGdV_mC4_/TYLCGdB from (A). This experiment was repeated three times with the same results. The error bar indicates the *SD*.

To further characterise the function of TYLCGdV C4 protein, a time‐course experiment was performed. At 7 dpi, *N. benthamiana* plants infected by TYLCGdV/TYLCGdB showed newly curled and mosaic leaves, whereas *N. benthamiana* plants infected by TYLCGdV_mC4_/TYLCGdB showed no obvious symptoms compared with plants agroinfiltrated with EV (Figure 5A). At 14 dpi, *N. benthamiana* plants infected by TYLCGdV_mC4_/TYLCGdB showed newly curled and mosaic leaves, but the symptoms were milder than those of TYLCGdV/TYLCGdB‐infected plants (Figure 5A). However, at 25 dpi, the symptoms in TYLCGdV_mC4_/TYLCGdB‐infected plants were more severe than those in TYLCGdV/TYLCGdB‐infected plants (Figure 5A). Moreover, at 61 dpi, *N. benthamiana* plants infected by TYLCGdV_mC4_/TYLCGdB showed much more severe symptoms, including stem bending, compared with TYLCGdV/TYLCGdB‐infected plants (Figure S3). Consistent with the symptoms, qPCR detection also showed that the virus and betasatellite accumulation in TYLCGdV_mC4_/TYLCGdB was much lower than that in TYLCGdV/TYLCGdB at 7 and 14 dpi (Figure 5B,C). However, at 25 dpi, the accumulation of TYLCGdV_mC4_/TYLCGdB was much higher than that of TYLCGdV/TYLCGdB (Figure 5B,C).

These results imply that C4 may contribute to the virus infection at the early stage of infection but negatively regulates the late‐stage infection.

### 2.5 C4 Protein Is Not a Pathogenicity Determinant

**FIGURE 6:**
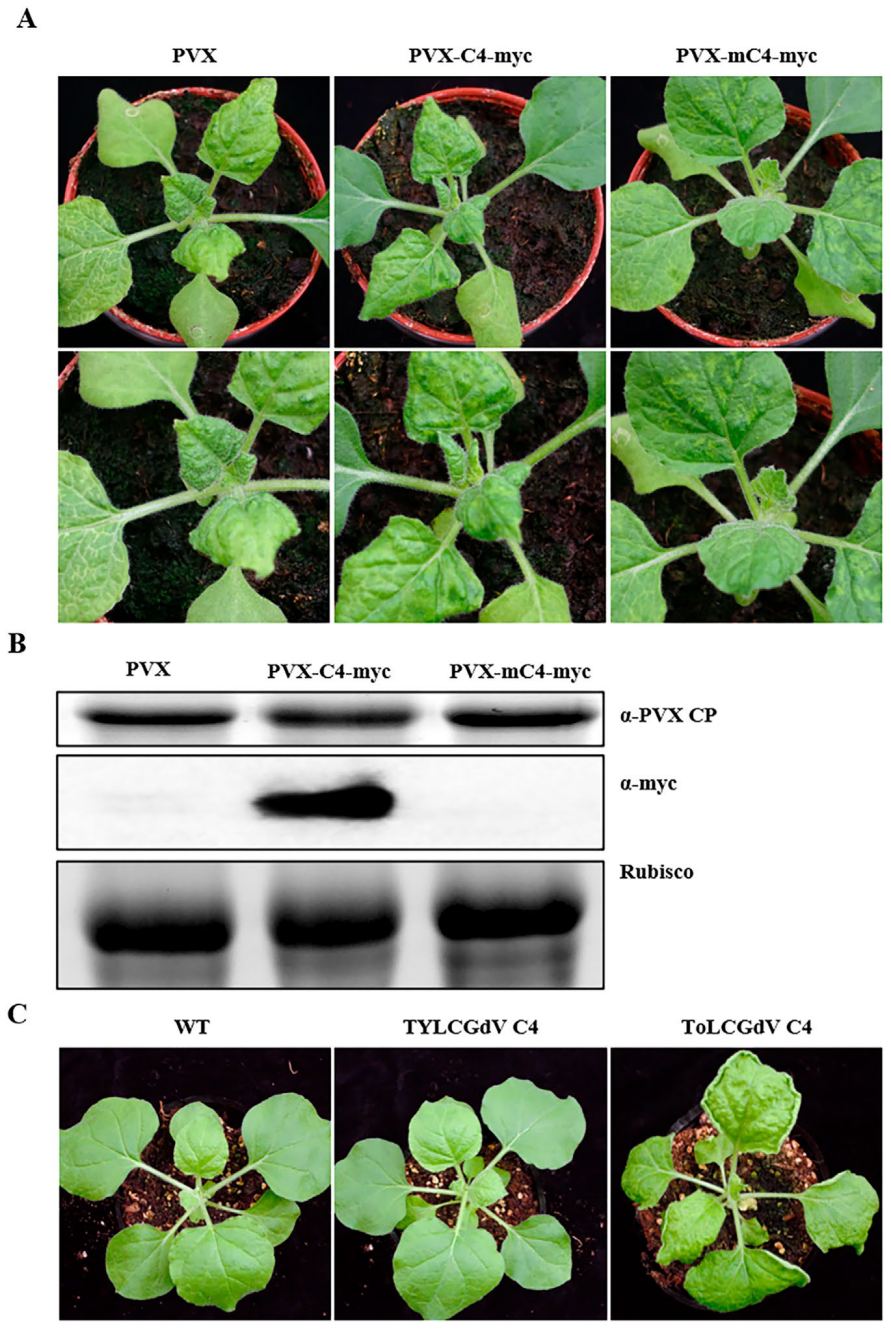
TYLCGdV C4 is not a pathogenic determinant. (A) Symptoms of *Nicotiana benthamiana* plants infected by PVX, PVX‐C4‐myc or PVX‐mC4‐myc at 9 days post‐inoculation. (B) Western blot detection of PVX coat protein (CP) accumulation and C4 expression in plants from (A) using anti‐PVX CP and anti‐myc antibodies, respectively. RuBisCO represents the same loading. (C) Comparison between the phenotypes of TYLCGdV C4 and ToLCGdV C4 transgenic *N. benthamiana* plants. Wild‐type (WT) plants were used as controls.

Many C4/AC4 proteins of geminiviruses act as pathogenicity determinants, potentially contributing to symptom development in plants that are either carrying the virus or expressing the C4 gene transgenically. TYLCGdV C4 was expressed by PVX vector (PVX‐C4‐myc) to investigate whether the C4 protein could influence symptom development in a heterologous viral system. PVX‐mC4‐myc, with a C4 start codon mutation, was also constructed as a control. *Agrobacterium* strains containing PVX, PVX‐C4‐myc or PVX‐mC4‐myc were infiltrated into *N. benthamiana* plants. All *N. benthamiana* plants showed obvious mosaic symptoms at 4 dpi and there was no difference among plants infected by PVX, PVX‐C4‐myc or PVX‐mC4‐myc (Figure S4). At 9 dpi, the disease symptoms of *N. benthamiana* plants infected by PVX, PVX‐C4‐myc or PVX‐mC4‐myc showed no visible difference (Figure 6A). PVX accumulation, detected by western blot using PVX CP antibody, also showed no significant difference (Figure 6B). Moreover, TYLCGdV C4 transgenic *N. benthamiana* plants showed no abnormal development compared with plants expressing ToLCGdV C4 (Figure 6C), a pathogenicity determinant described previously (Li et al. 2020).

These results demonstrate that TYLCGdV C4 may be not a pathogenicity determinant.

### 2.6 C4 Protein Does Not Suppress Local or Systemic PTGS

**FIGURE 7:**
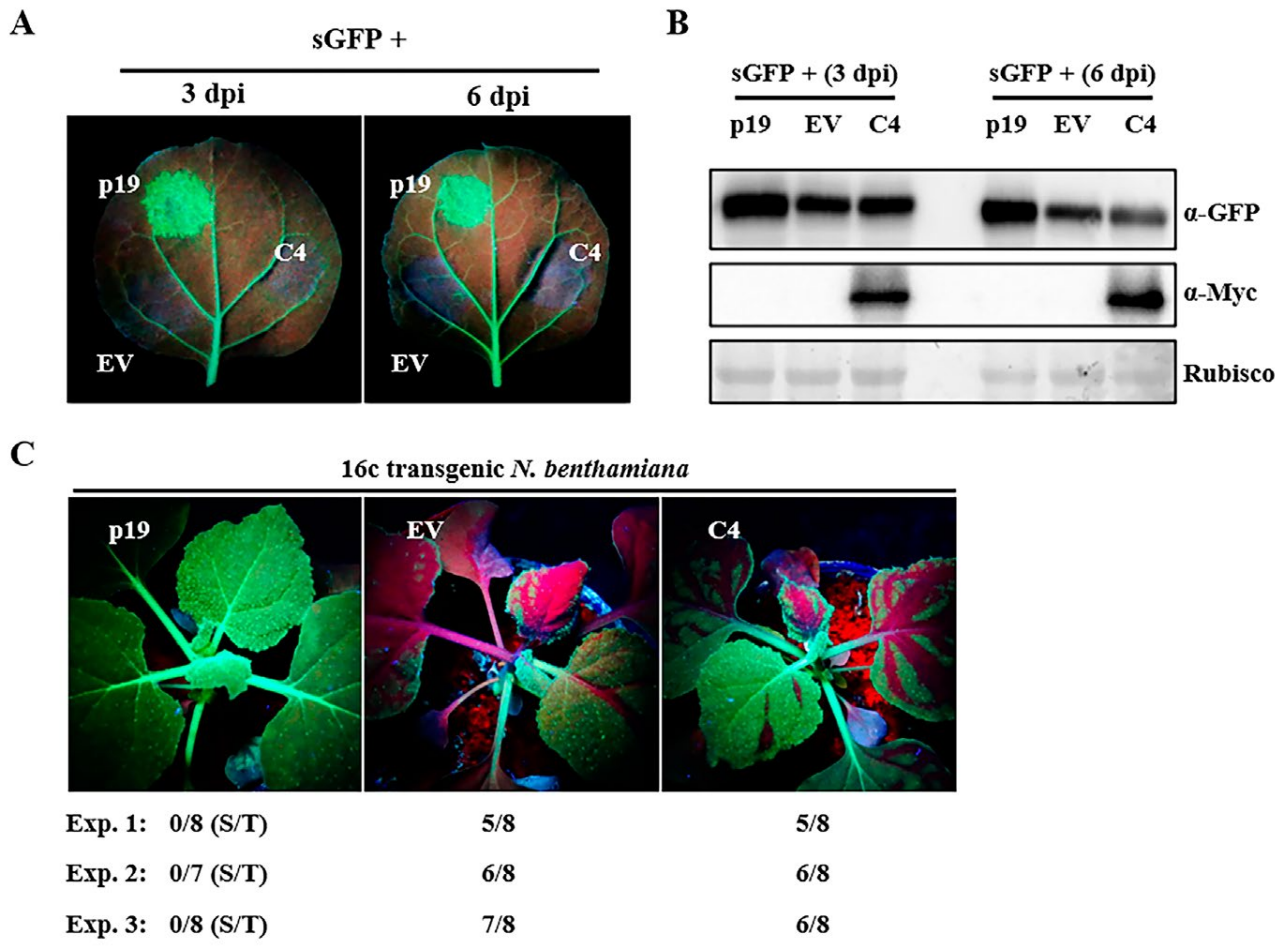
TYLCGdV C4 does not inhibit local and systemic post‐transcriptional gene silencing (PTGS). (A) Effect of TYLCGdV C4 on local PTGS. Wild‐type *Nicotiana benthamiana* plants were co‐infiltrated with *Agrobacterium* strains expressing C4 and sGFP, or p19 and sGFP (positive control), or empty vector (EV) and sGFP (negative control). GFP fluorescence was observed at 3 and 6 days post‐inoculation (dpi) using a long‐wave UV lamp. (B) Western blot detection of the green fluorescent protein (GFP) accumulation and the expression of TYLCGdV C4 in leaf patches from (A) at 3 and 6 dpi. RuBisCO represents the same loading. (C) Effect of TYLCGdV C4 on systemic PTGS. Transgenic 16c *N. benthamiana* plants were co‐infiltrated with *Agrobacterium* strains expressing C4 and sGFP, or p19 and sGFP (positive control), or EV and sGFP (negative control). GFP fluorescence was observed at 12 dpi with UV light. This experiment was repeated three times, as listed below the photographs. S/T indicates the systemic silencing plants (S) among the total infiltrated plants (T).

Geminiviral C4/AC4 proteins have been reported to be a viral suppressor of RNA silencing (VSR), which inhibits both PTGS and TGS (Vanitharani et al. 2004; Bisaro 2006; Gopal et al. 2007). Because TYLCGdV C4 is not a pathogenicity determinant, its RNA silencing suppression activity was tested using the *Agrobacterium* infiltration method (Johansen 2001). The *Agrobacterium* strain expressing positive‐sense *GFP* (sGFP) (Bragg and Jackson 2004) was co‐infiltrated into the leaves of WT *N. benthamiana* plants along with the strains expressing either C4, p19 or EV. At 3 and 6 dpi, strong green fluorescence was observed in leaf patches co‐infiltrated with p19 and sGFP (Figure 7A). However, no fluorescence was observed in regions co‐infiltrated with sGFP and EV, or sGFP and C4 (Figure 7A). Western blot detection showed that GFP accumulation in regions co‐infiltrated with sGFP and C4 was no higher than that in leaf patches co‐infiltrated with sGFP and EV (Figure 7B), suggesting that TYLCGdV C4 does not suppress local PTGS.

To further explore the systemic PTGS suppression activity of C4 protein, the combined *Agrobacterium* strains expressing sGFP+C4, sGFP+p19 or sGFP+EV were infiltrated into the leaves of 16c *N. benthamiana* plants that stably express GFP protein (Voinnet and Baulcombe 1997). GFP fluorescence was observed at 12 dpi. The results showed that the silencing signal was inhibited in transgenic plants co‐infiltrated with sGFP+p19 (Figure 7C). However, in transgenic plants co‐infiltrated with sGFP+C4 or sGFP+EV, GFP silencing could be observed from the infiltrated leaves to the upper leaves (Figure 7C). Thus, TYLCGdV C4 neither inhibits local PTGS nor systemic PTGS.

### 2.7 C4 Protein Does Not Suppress TGS

**FIGURE 8:**
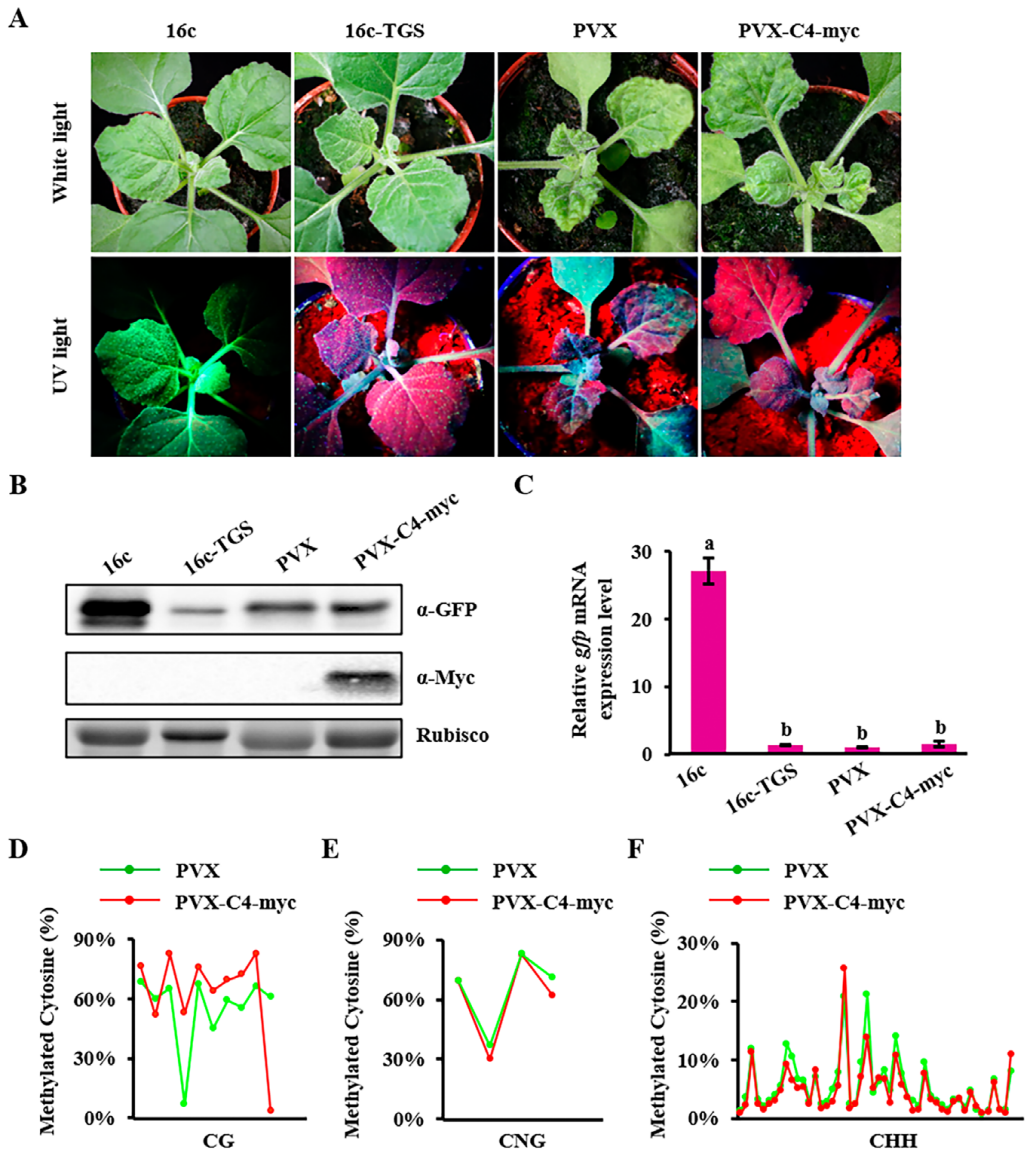
TYLCGdV C4 does not inhibit transcriptional gene silencing (TGS). (A) Suppression of TYLCGdV C4 on TGS. Symptoms of 16c‐TGS *Nicotiana benthamiana* plants agroinfiltrated with PVX or PVX‐C4‐myc at 12 days post‐inoculation (dpi). Photographs were taken under white light and UV light; 16c‐GFP transgenic plants and 16c‐TGS plants were used as positive and negative controls, respectively. (B) Western blot detection of GFP and C4 accumulation in plants from (A). RuBisCO was used as an equal loading control. (C) Reverse transcription‐quantitative PCR detection of *gfp* mRNA accumulation in plants from (A). Different letters indicate significant differences among different treatments. Cytosine methylation levels of CG (D), CNG (E) and CHH (F) in the 35S promoter region of *gfp* in 16c‐TGS plants infected by PVX or PVX‐C4‐Myc from (A). Green and red dots represent cytosine nucleotides in the 35S promoter region. The *x*‐axis indicates the location of the CG, CNG and CHH methylation sites. Detailed information on CG, CNG and CHH methylation levels is listed in Table S2.

TGS is one of the major pathways exploited by plants to counter DNA virus infection. To explore the TGS suppression activity of TYLCGdV C4, 16c‐TGS *N. benthamiana* plants (Buchmann et al. 2009; Raja, Wolf, and Bisaro 2010; Li et al. 2020) were agroinfiltrated with PVX or PVX‐C4‐myc. At 12 dpi, GFP fluorescence could be observed in 16c transgenic plants, but no visible fluorescence could be seen in 16c‐TGS plants or in plants infiltrated with PVX or PVX‐C4‐myc (Figure 8A). Moreover, C4 expression did not contribute to the GFP protein or *gfp* mRNA accumulation compared with PVX alone (Figure 8B,C). Next‐generation sequencing‐based bisulphite sequencing PCR was used to detect the cytosine methylation level of 35S promoter in *N. benthamiana* plants infiltrated with PVX or PVX‐C4‐myc. The results showed no remarkable difference in the cytosine methylation levels of CG, CNG and CHH (Figure 8D–F and Table S2). These results imply that TYLCGdV C4 does not suppress methylation‐mediated TGS.

### 2.8 C4 Modulates the DNA Methylation Level of TYLCGdV Genome at Different Stages of Infection

**FIGURE 9:**
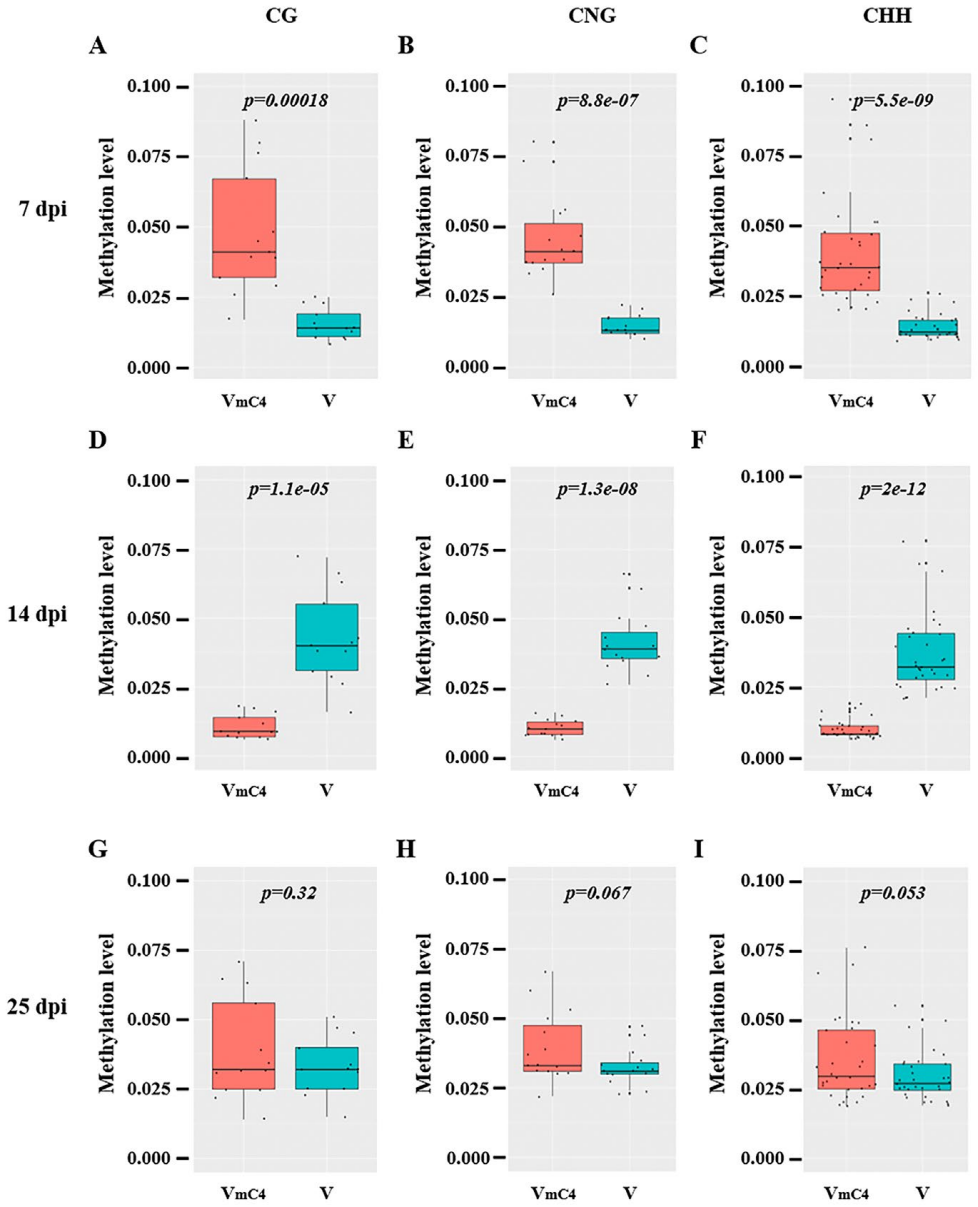
TYLCGdV C4 modulates the cytosine methylation levels of the viral genome at different infection stages. Wild‐typ*e Nicotiana benthamiana* plants were co‐infiltrated with TYLCGdV/TYLCGdB or TYLCGdV_mC4_ /TYLCGdB. The upper leaves were collected at 7, 14 and 25 days post‐inoculation (dpi) and subjected to next‐generation sequencing‐based bisulphite sequencing PCR analysis. The methylation level of TYLCGdV was assessed by analysing the cytosine methylation of CG, CNG and CHH sites in the region of TYLCGdV *AV1* (CP). V_mC4_, TYLCGdV_mC4_/TYLCGdB. V, TYLCGdV/TYLCGdB. Each box indicates the average methylation level of each group. A two‐tailed *t* test was used to identify significant differences between the two groups. Detailed cytosine methylation level of each site in the coat protein (*CP*) region are listed in Table S3. (A, B, and C) The cytosine methylation levels at CG, CNG, and CHH sites, respectively, for TYLCGdV_mC4_ and TYLCGdB at 7 dpi, (D, E, and F) at 14 dpi, and (G, H, and I) at 25 dpi.

Previous studies have demonstrated that DNA methylation of the geminivirus genome attenuates viral virulence (Zarreen and Chakraborty 2020). To test the effect of the C4 protein on the DNA methylation level of the TYLCGdV genome, total DNA was extracted from *N. benthamiana* plants infected by TYLCGdV/TYLCGdB or TYLCGdV_mC4_/TYLCGdB at 7, 14, and 25 dpi. Then, next‐generation sequencing‐based bisulphite sequencing PCR was used to examine the cytosine methylation level of the TYLCGdV *AV1* gene. At 7 dpi, the cytosine methylation levels of CG, CNG and CHH sites in the TYLCGdV_mC4_ *AV1* gene in the upper leaves are significantly higher than those in the TYLCGdV *AV1* gene (Figure 9A–C). However, at 14 dpi, the cytosine methylation level of the TYLCGdV_mC4_ *AV1* gene becomes lower than that of the TYLCGdV *AV1* gene (Figure 9D–F). Surprisingly, at 25 dpi, the cytosine methylation levels of TYLCGdV *AV1* and TYLCGdV_mC4_ *AV1* show no distinguishable difference (Figure 9G–I). These results suggest that TYLCGdV C4 may inhibit the DNA methylation of the viral genome at the early stages of infection and contribute to the DNA methylation in the middle and late stages of infection.

## 3 Discussion

In this study, we found that although TYLCGdV alone could infect *N. benthamiana*, it requires betasatellite to cause significant symptoms. Monopartite begomoviruses are often associated with circular ssDNA satellites, namely alphasatellites, betasatellites and deltasatellites (Zhou 2013; Varsani et al. 2021). Many monopartite begomoviruses from the OW are often associated with betasatellites (Zhou 2013; Mubin et al. 2019), and in most cases, betasatellites significantly enhance the infection of their helper viruses (Yang et al. 2019). Betasatellites encode a well‐known protein, βC1, that is essential for typical symptom development and functions as a VSR by inhibiting both PTGS and TGS (Li, Yang, et al. 2018; Shukla, Dalal, and Malathi 2013; Yang, Xie, et al. 2011; Yang, Guo, et al. 2011). We found that disruption of βC1 expression significantly reduced both virus accumulation and symptom development, demonstrating that βC1 plays an important role in TYLCGdV infection. However, compared to TYLCGdV‐infected plants, disease symptom were still visible in plants infected by TYLCGdV/TYLCGdB_mβC1_, implying that there may be another protein encoded by betasatellite that could also contribute to TYLCGdV infection. Recently, a novel protein, βV1, encoded by the virion‐sense strand of betasatellite, was identified (Hu et al. 2020; Gupta et al. 2022). Disruption of βV1 expression reduced both the virulence and viral load of tomato yellow leaf curl China virus (TYLCCNV), suggesting that βV1 also contributes to begomovirus infection (Hu et al. 2020). We also performed ORF prediction using SnapGene and the NCBI ORF finder tool (https://www.ncbi.nlm.nih.gov/orffinder/), setting a threshold of at least 45 nucleotides. This analysis revealed that the TYLCGdB genome contains up to 20 ORFs exceeding this length. Notably, TYLCGdB also harbours an ORF on the virion‐sense strand that is analogous to the βV1 protein of TYLCCNB, suggesting that this protein may also play a role in TYLCGdV infection.

The phylogenetic tree analysis showed that the TYLCGdV C4 protein shares the highest amino acid identity with C4 proteins of ToLCVV (91.75%), ToLCHnV (89.69%), TbLCTHV (87.63%) and TYLCCNV (85.57%). However, TYLCGdV C4 shares relatively lower similarity with the C4 protein of ToLCGdV (42.27%), TYLCYnV (49.48%), and TYLCV (68.04%). Recombination is one of the major forces that drive the evolution of begomoviruses, leading to the virus being more adaptable to new environments. Recombination between TYLCV and TYLCSV results in two new begomoviruses in the Mediterranean Basin, the tomato yellow leaf curl Málaga virus (TYLCMalV) and the tomato yellow leaf curl Axarquía virus (TYLCAxV), with better ecological fitness and a broader host range (Fiallo‐Olivé and Navas‐Castillo 2023; Fiallo‐Olivé et al. 2019; Monci et al. 2002; García‐Andrés et al. 2006; Belabess et al. 2015, 2016). Tomato leaf curl Yunnan virus (TLCYnV), a begomovirus initially isolated from *Malvastrum coromandelianum* , is believed to have evolved from tomato yellow leaf curl China virus (TYLCCNV) through recombination. This evolution has endowed TLCYnV with increased virulence, conferred by the acquisition of a more potent C4 gene, thereby rendering it highly infectious to a broad spectrum of host plants (Xie et al. 2013). Based on this, we propose that TYLCGdV is a recombinant begomovirus that likely originated from Southeast Asian begomoviruses.

In our study, we found that disruption of C4 protein expression affected TYLCGdV accumulation in *N. benthamiana* plants. Further study demonstrated that the accumulation of the mutant virus without C4 expression was lower at the early infection stage but higher at the late stage compared to the WT virus. C4/AC4 is one of the smallest and most variable proteins among begomoviruses, with the amino acid length of C4/AC4 ranging from 58 to 140 residues (Kumar and Dasgupta 2023), indicating the function of C4/AC4 may also be diverse (Medina‐Puche et al. 2021). Generally, most of the C4/AC4 proteins play an important role during begomovirus infection, including functioning as pathogenicity determinants and inducing disease‐like symptoms in transgenic plants (Latham et al. 1997; Mills‐Lujan and Deom 2010; Park et al. 2010; Mills‐Lujan et al. 2015; Gao et al. 2022). They also suppress PTGS and TGS (Kon, Sharma, and Ikegami 2007; Luna et al. 2012; Rosas‐Diaz et al. 2018; Ismayil et al. 2018), inhibit HR (Mei et al. 2020), suppress plant hormone signalling immune pathways (Medina‐Puche et al. 2021, 2020; Zhao, Ji, et al. 2024), and increase drought tolerance in host plants (Corrales‐Gutierrez et al. 2020). Although the AC4 proteins may not be functional in some bipartite begomoviruses (Pooma and Petty 1996; Hong and Stanley 1995; Hoogstraten, Hanson, and Maxwell 1996), there are no reports indicating that the C4 protein does not play a role in monopartite begomovirus infection. Therefore, it is intriguing that the C4 protein of TYLCGdV, a monopartite begomovirus associated with a betasatellite, functions differently from other begomovirus C4/AC4 proteins.

RNA‐directed DNA methylation is one of the major antiviral defence pathways for plants against DNA viruses, as it involves de novo cytosine methylation of the viral genome to repress the transcription of DNA virus. We have demonstrated that TYLCGdV C4 protein modulates the cytosine methylation level of the TYLCGdV genome at different infection stages. We speculate that DNA methylation affects virus accumulation by inhibiting virus replication. At the early infection stage, the methylation level in *AV1* gene of mutant virus with C4 disruption is higher than that of WT virus, leading to the lower virus accumulation of mutant virus than WT virus. However, at the middle stage of infection, the methylation level of mutant virus is lower than that of WT virus, and this maybe the reason that the virus accumulation level of mutant virus is higher than that of WT virus at late infection stage. We also examined the cytosine methylation level in other regions like IR and the promoter of *AV1* gene, which showed no significant difference between the WT and mutant virus (Figure S5). TYLCGdV C4 neither suppressed PTGS nor TGS; thus, the relationship between the C4 protein and the methylation level of virus genome is still unknown. It is intriguing that, in the absence of betasatellite, the viral accumulation of TYLCGdV was higher than that of TYLCGdV_mC4_, suggesting that the modulation of C4 protein may be overshadowed by the presence of the betasatellite. The C4 protein may function by interacting with other TYLCGdV proteins or host proteins. Recent research has shed light on the intricate network of intraviral protein–protein interactions during virus infection. The interaction between viral proteins can modify the subcellular localisation of the proteins involved. TYLCV CP has been found to interact with C2, and this interaction triggers the relocalisation of C2 protein from the nucleoplasm to the nucleolus. This research also indicated that TYLCV C4 can interact with C2 and C3, but the exact roles and implications of these interactions are not yet fully understood and require further investigation (Wang et al. 2022). It is suggested that, during a viral infection, C4 of TYLCGdV may also engage in interactions with other viral proteins, which could potentially alter its subcellular distribution and, consequently, its functional capabilities. In addition, we also speculate that the expression level of C4 protein may vary at different infection stages. As expected, the level of C4 expression was relatively low at the early infection stage, and higher at middle and late stages (Figure S6). Hence, future research may focus on identifying viral or host proteins that interact with TYLCGdV C4.

## 4 Experimental Procedures

### 4.1 Plasmid Construction

To construct TYLCGdV infectious clone containing a 1.3‐mer tandem repeat, the full‐length and 0.8 kb fragments of TYLCGdV were amplified using primer pairs TYLCGdV‐1.0A‐F1/R1 and TYLCGdV‐0.3A‐F2/R2 (Table S1), respectively. Then, the products were introduced into linearised pGreenII (Hellens et al. 2000) to make pGreenII‐TYLCGdV (Figure S1) using Seamless Assembly Cloning kit (TransGen Biotech). To generate pGreenII‐TYLCGdV_mC4_, the mutation of ATG to ACG in the start codon of C4 ORF was introduced by PCR with the primer pair mC4‐F/R (Table S1). Notably, the mutation in C4 has no effect on the expression of Rep protein. The infectious clone of TYLCGdB, which contains a 1.5‐mer tandem repeat (Figure S1), was introduced into pGreenII using TYLCGdB‐1.0A‐F1/R1 and TYLCGdB‐0.5A‐F2/R2 (Table S1) primer pairs, following the same strategy. To generate the mutant virus with disrupted βC1 expression, the 1.5‐mer tandem repeat sequence of the betasatellite, with mutations in the methionine residues located at positions 1, 9 and 16, was produced by gene synthesis. Specifically, the start codon ATG was altered to ACG to abolish βC1 protein expression. Subsequently, the modified betasatellite was cloned into the pGreenII vector using the Seamless Assembly Cloning kit, with the TYLCGdB‐1.0A‐F1/TYLCGdB‐0.5A‐R2 primer pair, as detailed in Table S1. The mutations in C4 and βC1 were confirmed by sequencing (Figure S7).

To make pGD‐C4‐myc and pGD‐mC4‐myc, the nucleotide sequences of C4 and mC4 were amplified from pGreenII‐TYLCGdV and pGreenII‐TYLCGdV_mC4_, respectively, and inserted into XhoI‐ and BamHI‐digested pGD‐myc (Goodin et al. 2002). PVX‐C4‐myc and PVX‐mC4‐myc were obtained by introducing C4‐myc and mC4‐myc fragments into pGR107 (Lu et al. 2003).

### 4.2 Plant Growth Conditions

*Nicotiana benthamiana* plants were grown in a controlled growth chamber under long‐day conditions (13 h light/11 h dark) at 24°C.

### 4.3 Plant Materials and *Agrobacterium* Infiltration

Wild‐type *N. benthamiana* plants are preserved by our laboratory. The 16c transgenic line expressing GFP protein was provided by Dawei Li's laboratory at China Agricultural University (CAU). The 16c‐TGS plants were generated in our laboratory according to the method published previously (Buchmann et al. 2009; Raja, Wolf, and Bisaro 2010). Briefly, TRV virus‐induced gene silencing (VIGS) vector containing a fragment of 35S promoter sequence was infiltrated into the 16c transgenic plants. After waiting until flowering, GFP‐silenced flowers were selected using a long‐wave UV lamp. Under the long‐wave UV lamp, the silenced flowers appear red, whereas the non‐silenced flowers show green fluorescence. The silenced flowers were marked, and seeds generated from these silenced flowers were collected.

Transgenic *N. benthamiana* plants expressing TYLCGdV C4 or ToLCGdV C4 were generated in this study. In brief, TYLCGdV C4‐myc or ToLCGdV C4‐myc was amplified and inserted into pBWA(V)HS. The generation of transgenic plants was performed according to the method reported previously (Xie et al. 2013).

The infectious clones were transformed into *Agrobacterium tumefaciens* GV3101 according to the manufacturer's instructions. For virus inoculation, *Agrobacterium* strains containing the infectious clones were infiltrated into 5‐ to 6‐leaf stage *N. benthamiana* plants. For PTGS assays, *Agrobacterium* cultures (OD_600_ = 0.5) harbouring plasmids expressing positive‐sGFP (Bragg and Jackson 2004) and *Agrobacterium* strain (OD_600_ = 0.5) harbouring pGD‐C4‐myc were co‐infiltrated into WT and 16c transgenic *N. benthamiana* plants. The p19 encoded by tomato bushy stunt virus (Voinnet, Pinto, and Baulcombe 1999) and EV were used as positive and negative controls, respectively. For TGS assays, *Agrobacterium* cultures (OD_600_ = 0.5) harbouring plasmids expressing PVX‐C4 or PVX‐mC4 was infiltrated into 16c‐TGS *N. benthamiana* plants.

### 4.4 Viral Accumulation Detection

Total DNA of the viral samples was extracted using the CTAB method (Doyle and Doyle 1987), and the DNA of each sample was diluted to nearly the same concentration before qPCR. TYLCGdV‐qPCR‐F/TYLCGdV‐qPCR‐R and β‐qPCR‐F/β‐qPCR‐R (Table S1) were used for virus and betasatellite detection, respectively. Total RNA was extracted with TRIzol reagent (TaKaRa) and diluted to the same concentration. RNA was treated with RNase‐free rDNase I (TransGen Biotech) to remove DNA contamination before reverse transcription using PrimeScript RT Master Mix (TaKaRa). Real‐time PCR was performed with the Bio‐Rad CFX96 real‐time system using TB Green Premix Ex Taq (TaKaRa). For western blot detection, leaves were collected and weighed in equal amounts, followed by the protocol described previously (Li et al. 2020).

### 4.5 Phylogenetic Analyses

A phylogenetic tree was constructed using C4/AC4 protein sequences acquired from the NCBI database with MEGA 7 software (Kumar, Stecher, and Tamura 2016), employing the neighbour‐joining method.

### 4.6 Next‐Generation Sequencing‐Based Bisulphite Sequencing PCR

TGS suppression activity of the C4 protein was analysed by detecting the cytosine methylation level of the 35S promoter region in 16c‐TGS *N. benthamiana* plants. PCR products amplified from the TYLCGdV CP ORF with the CP‐BSP‐F1/R1 and CP‐BSP‐F2/R2 (Table S1) primer pairs were used to assess the methylation level of TYLCGdV *AV1*. The next‐generation sequencing‐based bisulphite sequencing PCR method was described previously (Li et al. 2020; Gao et al. 2014; Pan et al. 2018; Lister et al. 2009).

## Conflicts of Interest

The authors declare no conflicts of interest.

## Supporting information


**Figure S1:** Schematic presentation of the infectious clones of TYLCGdV and TYLCGdB.

**Figure S2:** Amino acid similarity comparison of ToLCGdV C4 and TYLCGdV C4.

**Figure S3:** Symptom comparison of the *Nicotiana benthamiana* plants infected by TYLCGdV/TYLCGdB and TYLCGdVmC4/TYLCGdB, respectively.

**Figure S4:** Symptoms of *Nicotiana benthamiana* plants infected by PVX‐C4‐Myc and PVX‐mC4‐Myc at different time points.

**Figure S5:** The cytosine methylation level in IR and V1P regions of ToLCGdV at different infection stages. IR, intergenic region; V1P, the promoter region of V1 protein.

**Figure S6:** The mRNA accumulation of C4 at different times.

**Figure S7:** Verification of the C4 and βC1 mutation by sequencing.

**Table S1:** Primers used in this study.

**Table S2:** Cytosine methylation level of each CG, CNG and CHH site in the 35S promoter region under PVX and PVX‐C4‐Myc infection.

**Table S3:** Cytosine methylation level of each CG, CNG and CHH site in the CP region of TYLCGdV or TYLCGdV_mC4_.

## Data Availability

The data that support the findings of this study are available from the corresponding author upon reasonable request.
