# Supplementary material for: Characterisation of a Betasatellite Associated With Tomato Yellow Leaf Curl Guangdong Virus and Discovery of an Unusual Modulation of Virus Infection Associated With C4 Protein
Source: Mol Plant Pathol. 2025 Jan 14;26(1):e70051. doi: 10.1111/mpp.70051 (PMC11732742; doi:10.1111/mpp.70051)
Supplement: Supplementary file 1 — Figure S1: Schematic presentation of the infectious clones of TYLCGdV and TYLCGdB. [file MPP-26-e70051-s001.pdf]

A

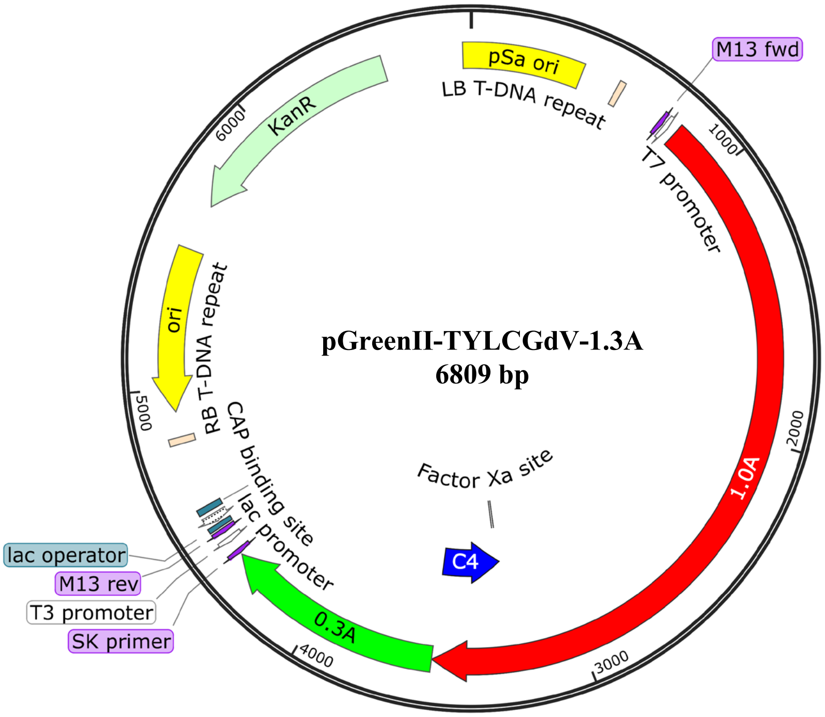

B

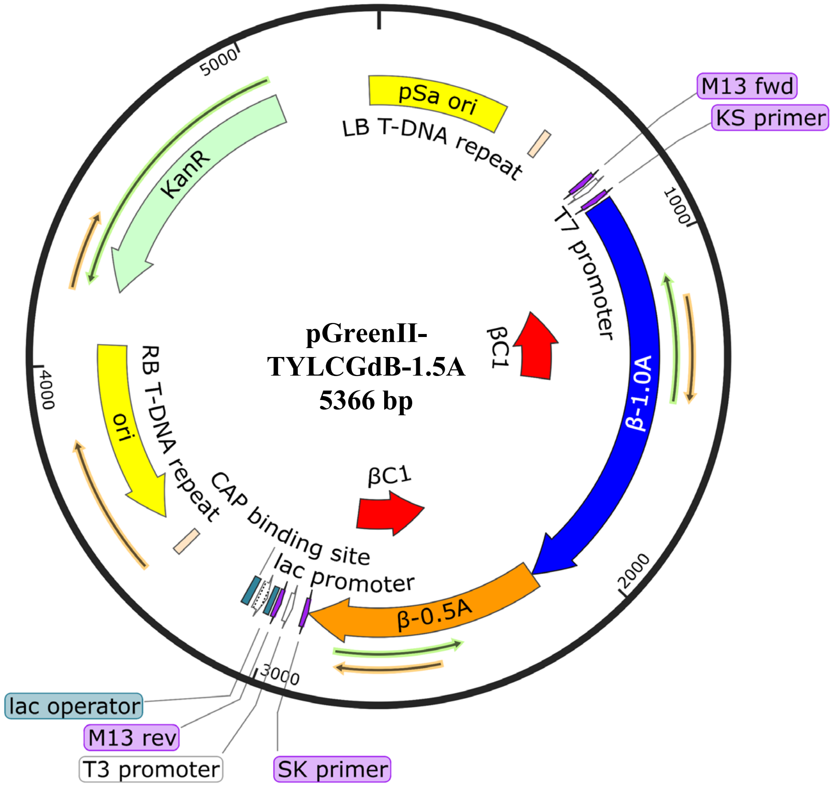

Supplemental Figure S1: Schematic presentation of the infectious clones of TYLCGdV and TYLCGdB
