# Supplementary material for: Characterisation of a Betasatellite Associated With Tomato Yellow Leaf Curl Guangdong Virus and Discovery of an Unusual Modulation of Virus Infection Associated With C4 Protein
Source: Mol Plant Pathol. 2025 Jan 14;26(1):e70051. doi: 10.1111/mpp.70051 (PMC11732742; doi:10.1111/mpp.70051)
Supplement: Supplementary file 2 — Figure S2: Amino acid similarity comparison of ToLCGdV C4 and TYLCGdV C4. [file MPP-26-e70051-s002.pdf]

Identity=42.27%

```
ToLCGdV C4  MGTLISTCLCNSKANTTARITDSSTWFPQPDQHISIRTFRELNPAPMSSP 50
TYLCGdV C4  MGLLTCISSSNSKESSNAKTTDSSISYPQPGQHISIRTFRALRAQQMLSP 50

ToLCGdV C4  TSIRMETSLNGVNSRSTDEVLGEAARMLTTHVQRL............  85
TYLCGdV C4  TWKKTETCLIMEFSRSMEDRLEEVANLPTTHMPRQSIQGPKLRPSIY  97
```

Supplemental Figure S2: Amino acid similarity comparison of ToLCGdV C4 and TYLCGdV C4.
