# Supplementary material for: Characterisation of a Betasatellite Associated With Tomato Yellow Leaf Curl Guangdong Virus and Discovery of an Unusual Modulation of Virus Infection Associated With C4 Protein
Source: Mol Plant Pathol. 2025 Jan 14;26(1):e70051. doi: 10.1111/mpp.70051 (PMC11732742; doi:10.1111/mpp.70051)
Supplement: Supplementary file 3 — Figure S3: Symptom comparison of the Nicotiana benthamiana plants infected by TYLCGdV/TYLCGdB and TYLCGdVmC4/TYLCGdB, respectively. [file MPP-26-e70051-s010.pdf]

TYLCGdV/TYLCGdB **TYLCGdV$_{mC4}$/TYLCGdB**

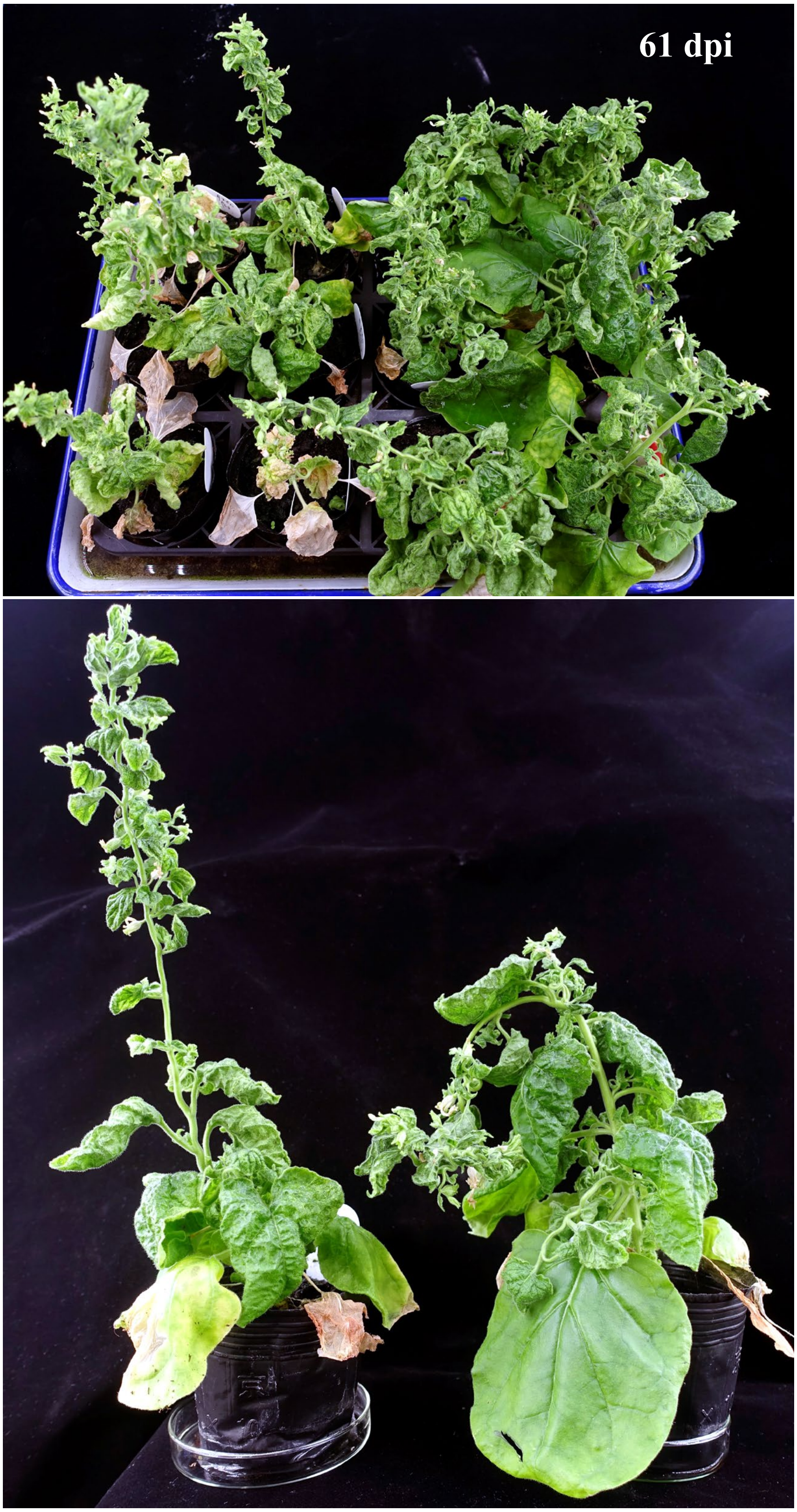

Supplemental Figure S3: Symptom comparison of the *N. benthamiana* plants infected by TYLCGdV/TYLCGdB and TYLCGdV$_{mC4}$/TYLCGdB, respectively.
