# Supplementary material for: Characterisation of a Betasatellite Associated With Tomato Yellow Leaf Curl Guangdong Virus and Discovery of an Unusual Modulation of Virus Infection Associated With C4 Protein
Source: Mol Plant Pathol. 2025 Jan 14;26(1):e70051. doi: 10.1111/mpp.70051 (PMC11732742; doi:10.1111/mpp.70051)
Supplement: Supplementary file 4 — Figure S4: Symptoms of Nicotiana benthamiana plants infected by PVX‐C4‐Myc and PVX‐mC4‐Myc at different time points. [file MPP-26-e70051-s009.pdf]

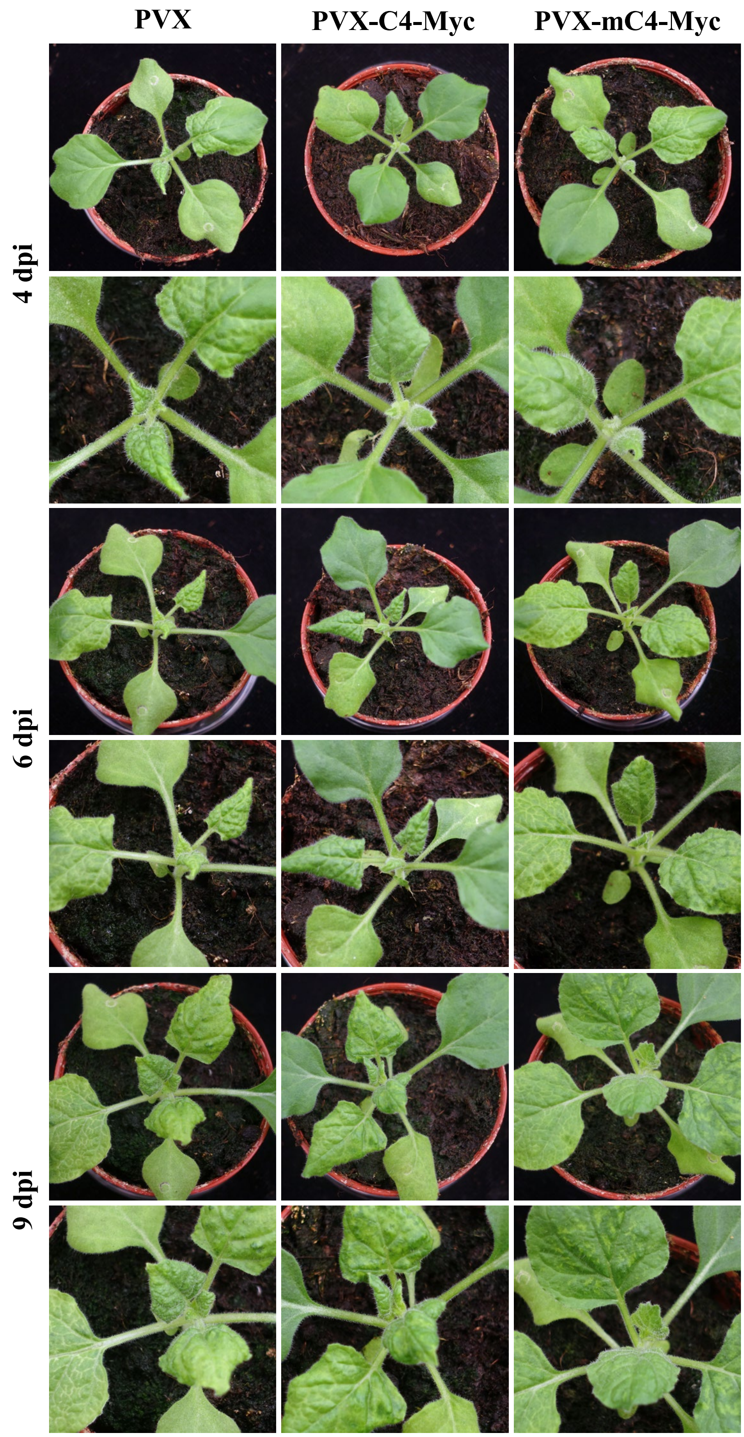

Supplemental Figure S4: Symptom of *N. benthamiana* plants infected by PVX-C4-Myc and PVX-mC4-Myc at different time points.
