# Supplementary material for: Characterisation of a Betasatellite Associated With Tomato Yellow Leaf Curl Guangdong Virus and Discovery of an Unusual Modulation of Virus Infection Associated With C4 Protein
Source: Mol Plant Pathol. 2025 Jan 14;26(1):e70051. doi: 10.1111/mpp.70051 (PMC11732742; doi:10.1111/mpp.70051)
Supplement: Supplementary file 5 — Figure S5: The cytosine methylation level in IR and V1P regions of ToLCGdV at different infection stages. IR, intergenic region; V1P, the promoter region of V1 protein. [file MPP-26-e70051-s004.pdf]

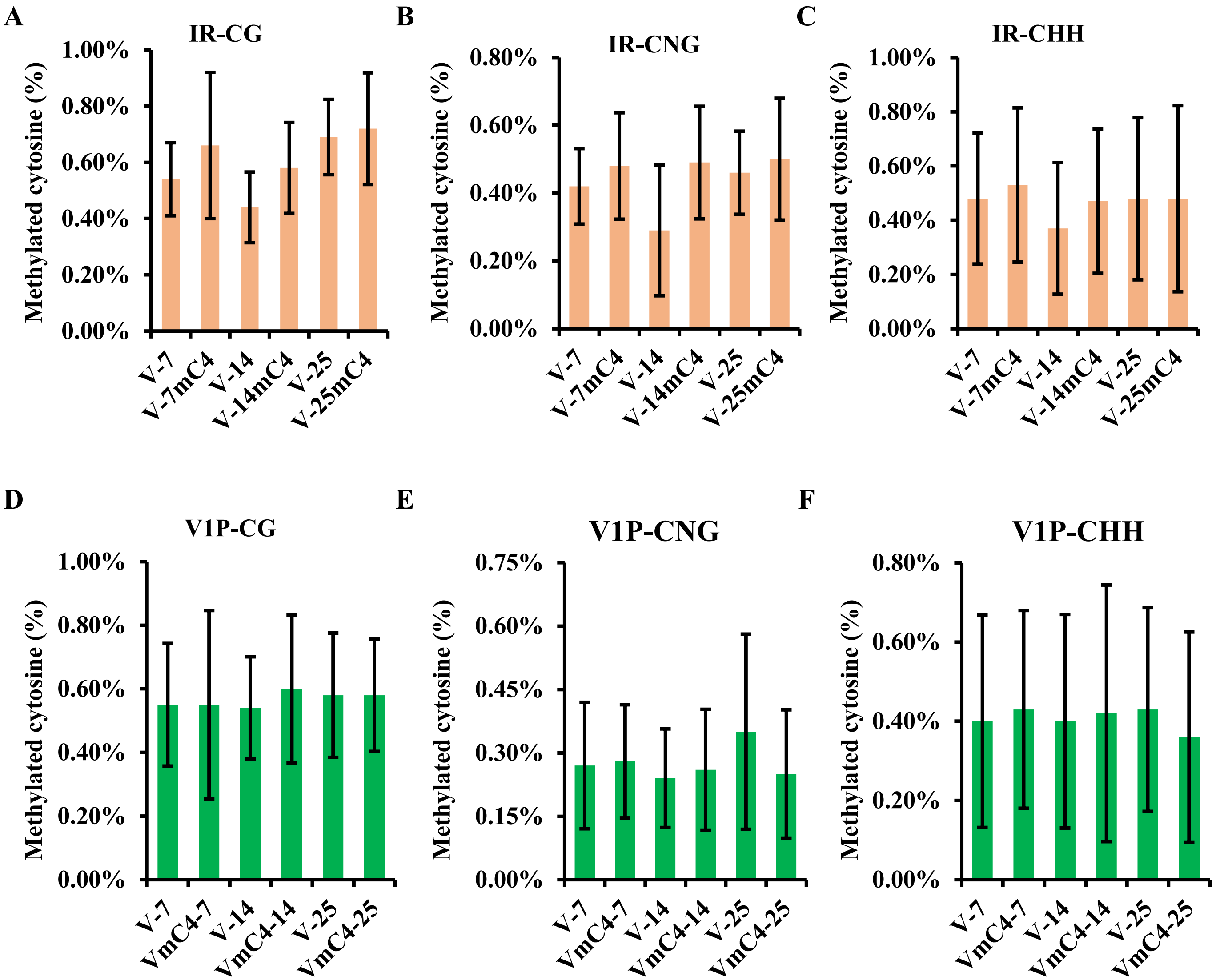

Supplemental Figure S5: The cytosine methylation level in IR and V1P regions of ToLCGdV at different infection stages. IR, intergenic region. V1P, the promoter region of V1 protein.
