# Supplementary figures and images for: Characterisation of a Betasatellite Associated With Tomato Yellow Leaf Curl Guangdong Virus and Discovery of an Unusual Modulation of Virus Infection Associated With C4 Protein
Source: Mol Plant Pathol. 2025 Jan 14;26(1):e70051. doi: 10.1111/mpp.70051 (PMC11732742; doi:10.1111/mpp.70051)

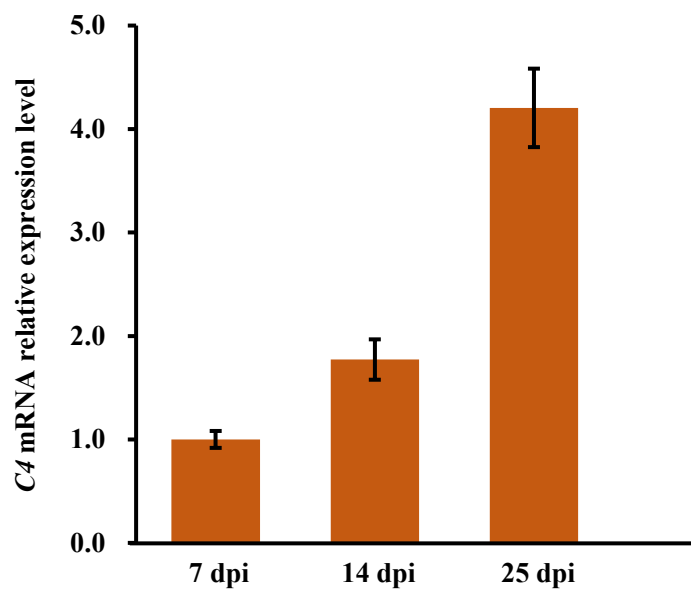

**Supplemental Figure S6: The mRNA accumulation of C4 in different times.**

Supplement: Supplementary file 6 — Figure S6: The mRNA accumulation of C4 at different times. [file MPP-26-e70051-s003.pdf]
