# Supplementary material for: Characterisation of a Betasatellite Associated With Tomato Yellow Leaf Curl Guangdong Virus and Discovery of an Unusual Modulation of Virus Infection Associated With C4 Protein
Source: Mol Plant Pathol. 2025 Jan 14;26(1):e70051. doi: 10.1111/mpp.70051 (PMC11732742; doi:10.1111/mpp.70051)
Supplement: Supplementary file 7 — Figure S7: Verification of the C4 and βC1 mutation by sequencing. [file MPP-26-e70051-s005.pdf]

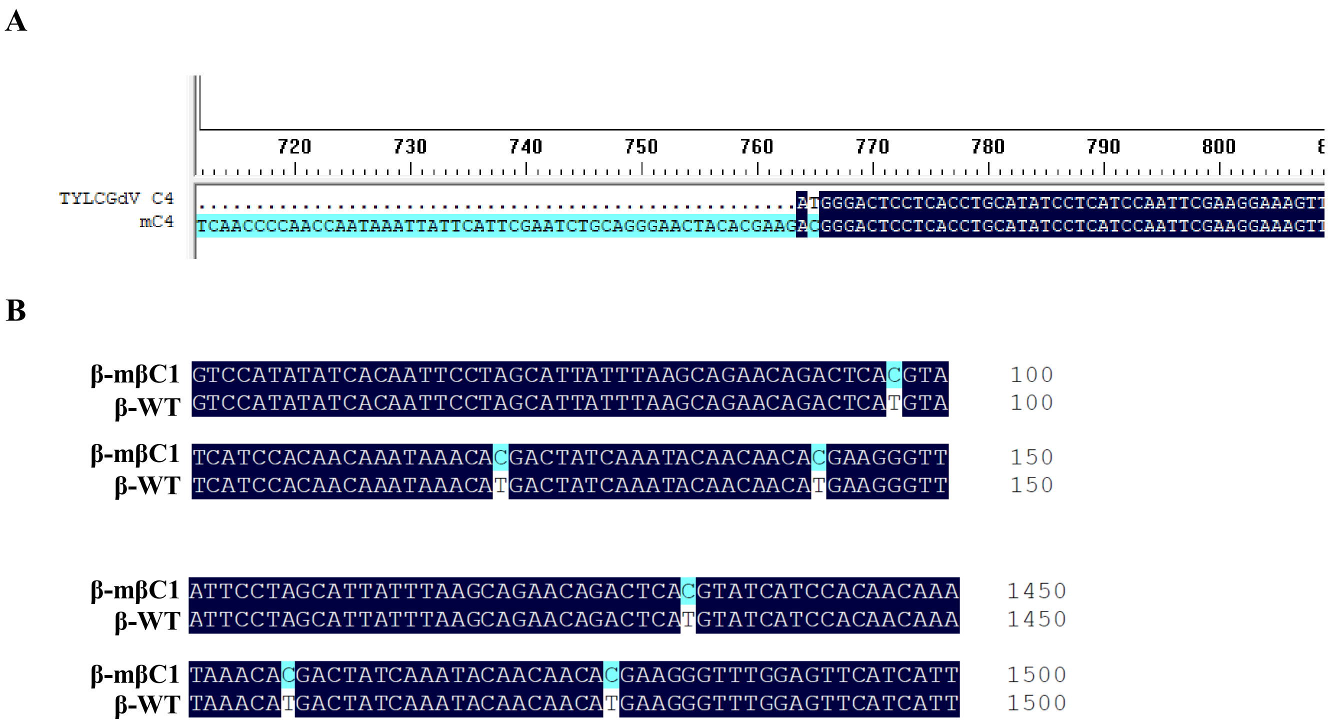

Supplemental Figure S7: Verification of the C4 and βC1 mutation by sequencing
