# Supplementary material for: Characterisation of a Betasatellite Associated With Tomato Yellow Leaf Curl Guangdong Virus and Discovery of an Unusual Modulation of Virus Infection Associated With C4 Protein
Source: Mol Plant Pathol. 2025 Jan 14;26(1):e70051. doi: 10.1111/mpp.70051 (PMC11732742; doi:10.1111/mpp.70051)
Supplement: Supplementary file 8 — Table S1: Primers used in this study. [file MPP-26-e70051-s007.docx]

# Supplementary Table S1 | Primers used in this study

| **Primer** | **Primer sequence (5**'**-3**'**) ^a, b^** | **Purpose** |
| --- | --- | --- |
| **TYLCGdV-1.0A-F1** | CCGGGCCCCCCCTCGAGCTTCGTGTAGTTCCCTGCAGA | pGreenII-1.3A-TYLCGdV |
| **TYLCGdV-1.0A-R1** | AGGGAACTACACGAAGATGGGACTCCTCACCT |  |
| **TYLCGdV-0.3A-F2** | GGTGAGGAGTCCCATCTTCGTGTAGTTCCCTGCAG |  |
| **TYLCGdV-0.3A-R2** | AGTGGATCCCCCGGGTTCACAACCTCGAGGAACAT |  |
| **TYLCGdB-1.0A-F1** | GTATCGATAAGCTTGAAACCACTACGCTA | pGreenII-1.5A-TYLCGdB |
| **TYLCGdB-1.0A-R1** | TAGCGTAGTGGTTTCTACCCTCCCAGGGGTA |  |
| **TYLCGdB-0.5A-F2** | TACCCCTGGGAGGGTAGAAACCACTACGCTA |  |
| **TYLCGdB-0.5A-R2** | AGAACTAGTGGATCCATCTCCACTAGTAGGCA |  |
| **mC4-F** | GGTGAGGAGTCCC*G*TCTTCGTGTAGTTC | pGreenII-1.3A-TYLCGdV_mC4_ |
| **mC4-R** | GAACTACACGAAGA*C*GGGACTCCTCACC |  |
| **pGD-C4-Myc-F** | CCGCTCGAGATGGGACTCCTCACCTGCAT | pGD-C4-Myc  pGD-mC4-Myc |
| **pGD-C4-Myc-R** | CGGGATCCATATATTGAGGGCCGCAGCTTT |  |
| **pGD-mC4-Myc-F** | CCGCTCGAGACGGGACTCCTCACCTGCAT |  |
| **PVX-C4-Myc -F** | CC**ATCGAT**ATGGGACTCCTCACCTGCAT | PVX-C4-Myc  PVX-mC4-Myc |
| **PVX-C4-Myc-R** | TTATGATCAGTTATCTAGATCCGGTGGATCTTTA |  |
| **PVX-C4-mMyc-F** | CC**ATCGAT**A*C*GGGACTCCTCACCTGCAT |  |
| **TYLCGdV-qPCR-F** | TGATAACGAGCCAAGTACAGCAACT | qPCR detection of virus accumulation |
| **TYLCGdV-qPCR-R** | CTTCGCAGCCTCTTGATGATTATACG |  |
| **β-qPCR-F** | ACAACAACATGAAGGGTTTGGAGTTC | qPCR detection of betasatellite accumulation |
| **β-qPCR-R** | TTCTTGGCTAATGCTGGTGACTTTG |  |
| **CP-BSP-F1** | GTTTYGTAGATATAGTTATTTTTATTTTYGTTTTTAAGG | Amplification of CP region for BSP analysis |
| **CP-BSP-R1** | CTTTTATACATCCTATACATCTTAAACTTTC |  |
| **CP-BSP-F2** | TATATTAATATTGTGATGTTTTTTTTAGTTYGTGATAGA |  |
| **CP-BSP-R2** | TACTCCTTACTTACATACTAACCACC |  |

^a^ Underlined and bold letters indicate the sequences of the restriction sites.

^b^ Underlined, italic, and bold letters indicate the sequences altered by site-specific mutagenesis
