# Supplementary material for: Characterisation of a Betasatellite Associated With Tomato Yellow Leaf Curl Guangdong Virus and Discovery of an Unusual Modulation of Virus Infection Associated With C4 Protein
Source: Mol Plant Pathol. 2025 Jan 14;26(1):e70051. doi: 10.1111/mpp.70051 (PMC11732742; doi:10.1111/mpp.70051)
Supplement: Supplementary file 9 — Table S2: Cytosine methylation level of each CG, CNG and CHH site in the 35S promoter region under PVX and PVX‐C4‐Myc infection. [file MPP-26-e70051-s008.docx]

# Table S2 | Cytosine methylation level of each CG, CNG, and CHH site in 35S promoter region under PVX and PVX-C4-Myc infection

|  | **PVX** | **PVX-C4-Myc** |
| --- | --- | --- |
| **Relative position of 35S promoter** | **Ratio of methylated cytosine** | **Ratio of methylated cytosine** |
| **CG** | | |
| **121** | 0.686 | 0.762 |
| **139** | 0.601 | 0.519 |
| **157** | 0.653 | 0.826 |
| **189** | 0.068 | 0.531 |
| **199** | 0.675 | 0.760 |
| **214** | 0.449 | 0.642 |
| **225** | 0.592 | 0.695 |
| **265** | 0.555 | 0.722 |
| **277** | 0.665 | 0.829 |
| **297** | 0.609 | 0.035 |
| **CNG** | | |
| **153** | 0.692 | 0.695 |
| **156** | 0.301 | 0.369 |
| **160** | 0.826 | 0.828 |
| **261** | 0.618 | 0.710 |
| **CHH** | | |
| **100** | 0.013 | 0.009 |
| **102** | 0.037 | 0.023 |
| **103** | 0.120 | 0.113 |
| **106** | 0.033 | 0.025 |
| **112** | 0.021 | 0.015 |
| **113** | 0.030 | 0.025 |
| **116** | 0.041 | 0.031 |
| **135** | 0.055 | 0.048 |
| **136** | 0.127 | 0.092 |
| **150** | 0.105 | 0.066 |
| **151** | 0.067 | 0.051 |
| **167** | 0.066 | 0.054 |
| **168** | 0.028 | 0.024 |
| **169** | 0.072 | 0.083 |
| **179** | 0.024 | 0.017 |
| **180** | 0.028 | 0.021 |
| **181** | 0.050 | 0.028 |
| **182** | 0.078 | 0.056 |
| **183** | 0.208 | 0.257 |
| **185** | 0.024 | 0.017 |
| **186** | 0.025 | 0.024 |
| **187** | 0.096 | 0.072 |
| **196** | 0.212 | 0.138 |
| **218** | 0.044 | 0.052 |
| **219** | 0.064 | 0.070 |
| **222** | 0.083 | 0.067 |
| **223** | 0.048 | 0.027 |
| **228** | 0.140 | 0.108 |
| **231** | 0.077 | 0.058 |
| **236** | 0.037 | 0.037 |
| **256** | 0.031 | 0.014 |
| **258** | 0.021 | 0.016 |
| **259** | 0.097 | 0.077 |
| **279** | 0.039 | 0.032 |
| **281** | 0.031 | 0.027 |
| **285** | 0.022 | 0.016 |
| **286** | 0.015 | 0.011 |
| **287** | 0.032 | 0.028 |
| **289** | 0.032 | 0.034 |
| **293** | 0.019 | 0.013 |
| **294** | 0.048 | 0.044 |
| **299** | 0.015 | 0.021 |
| **304** | 0.008 | 0.010 |
| **305** | 0.013 | 0.011 |
| **306** | 0.067 | 0.062 |
| **309** | 0.015 | 0.015 |
| **310** | 0.016 | 0.016 |
| **312** | 0.080 | 0.110 |
