# Supplementary material for: Characterisation of a Betasatellite Associated With Tomato Yellow Leaf Curl Guangdong Virus and Discovery of an Unusual Modulation of Virus Infection Associated With C4 Protein
Source: Mol Plant Pathol. 2025 Jan 14;26(1):e70051. doi: 10.1111/mpp.70051 (PMC11732742; doi:10.1111/mpp.70051)
Supplement: Supplementary file 10 — Table S3: Cytosine methylation level of each CG, CNG and CHH site in the CP region of TYLCGdV or TYLCGdVmC4. [file MPP-26-e70051-s006.docx]

# Table S3 | Cytosine methylation level of each CG, CNG, and CHH site in CP region of TYLCGdV or TYLCGdV_mC4_

**CG**

| **pos** | **V_mC4_-7** | **V-7** | **V_mC4_-14** | | **V-14** | **V_mC4_-25** | **V-25** |
| --- | --- | --- | --- | --- | --- | --- | --- |
| **164** | 0.032 | 0.011 | | 0.007 | 0.031 | 0.025 | 0.025 |
| **167** | 0.039 | 0.014 | | 0.009 | 0.038 | 0.031 | 0.031 |
| **170** | 0.041 | 0.014 | | 0.009 | 0.04 | 0.032 | 0.032 |
| **181** | 0.045 | 0.013 | | 0.008 | 0.043 | 0.034 | 0.034 |
| **200** | 0.039 | 0.014 | | 0.009 | 0.038 | 0.032 | 0.032 |
| **223** | 0.029 | 0.011 | | 0.007 | 0.029 | 0.025 | 0.025 |
| **254** | 0.026 | 0.01 | | 0.007 | 0.026 | 0.022 | 0.023 |
| **266** | 0.017 | 0.008 | | 0.006 | 0.016 | 0.014 | 0.015 |
| **638** | 0.067 | 0.019 | | 0.014 | 0.055 | 0.056 | 0.04 |
| **653** | 0.076 | 0.023 | | 0.017 | 0.063 | 0.063 | 0.045 |
| **656** | 0.088 | 0.025 | | 0.018 | 0.072 | 0.071 | 0.051 |
| **666** | 0.08 | 0.023 | | 0.016 | 0.066 | 0.065 | 0.047 |
| **676** | 0.048 | 0.016 | | 0.012 | 0.041 | 0.039 | 0.032 |

**CNG**

| **pos** | **V_mC4_-7** | **V-7** | **V_mC4_-14** | **V-14** | **V_mC4_-25** | **V-25** |
| --- | --- | --- | --- | --- | --- | --- |
| **169** | 0.038 | 0.013 | 0.008 | 0.037 | 0.03 | 0.03 |
| **184** | 0.042 | 0.012 | 0.008 | 0.04 | 0.033 | 0.032 |
| **196** | 0.041 | 0.012 | 0.008 | 0.039 | 0.033 | 0.032 |
| **199** | 0.037 | 0.013 | 0.008 | 0.036 | 0.031 | 0.031 |
| **204** | 0.037 | 0.012 | 0.008 | 0.036 | 0.031 | 0.031 |
| **207** | 0.035 | 0.012 | 0.008 | 0.035 | 0.03 | 0.03 |
| **216** | 0.026 | 0.01 | 0.006 | 0.026 | 0.022 | 0.023 |
| **563** | 0.055 | 0.018 | 0.013 | 0.05 | 0.053 | 0.038 |
| **575** | 0.045 | 0.017 | 0.012 | 0.043 | 0.045 | 0.033 |
| **609** | 0.033 | 0.013 | 0.01 | 0.029 | 0.033 | 0.024 |
| **615** | 0.038 | 0.013 | 0.01 | 0.033 | 0.037 | 0.027 |
| **631** | 0.056 | 0.018 | 0.013 | 0.047 | 0.05 | 0.035 |
| **652** | 0.08 | 0.022 | 0.016 | 0.066 | 0.067 | 0.047 |
| **669** | 0.073 | 0.021 | 0.015 | 0.061 | 0.06 | 0.044 |
| **675** | 0.047 | 0.015 | 0.011 | 0.04 | 0.039 | 0.03 |

**CHH**

| **pos** | **VmC4-7** | **V-7** | **VmC4-14** | **V-14** | **VmC4-25** | **V-25** |
| --- | --- | --- | --- | --- | --- | --- |
| **178** | 0.045 | 0.013 | 0.008 | 0.043 | 0.035 | 0.034 |
| **187** | 0.036 | 0.011 | 0.007 | 0.034 | 0.029 | 0.028 |
| **188** | 0.035 | 0.012 | 0.008 | 0.035 | 0.03 | 0.029 |
| **189** | 0.035 | 0.011 | 0.007 | 0.034 | 0.029 | 0.028 |
| **211** | 0.025 | 0.009 | 0.006 | 0.026 | 0.022 | 0.022 |
| **212** | 0.024 | 0.009 | 0.006 | 0.025 | 0.022 | 0.022 |
| **213** | 0.023 | 0.009 | 0.006 | 0.024 | 0.02 | 0.02 |
| **214** | 0.028 | 0.012 | 0.009 | 0.029 | 0.025 | 0.026 |
| **220** | 0.025 | 0.01 | 0.007 | 0.025 | 0.023 | 0.023 |
| **221** | 0.027 | 0.011 | 0.008 | 0.028 | 0.025 | 0.025 |
| **226** | 0.031 | 0.011 | 0.008 | 0.031 | 0.027 | 0.027 |
| **228** | 0.032 | 0.011 | 0.008 | 0.031 | 0.026 | 0.026 |
| **232** | 0.034 | 0.011 | 0.008 | 0.032 | 0.028 | 0.027 |
| **243** | 0.029 | 0.011 | 0.008 | 0.029 | 0.026 | 0.026 |
| **257** | 0.02 | 0.01 | 0.007 | 0.021 | 0.019 | 0.019 |
| **258** | 0.02 | 0.01 | 0.007 | 0.021 | 0.02 | 0.02 |
| **259** | 0.021 | 0.011 | 0.008 | 0.021 | 0.019 | 0.02 |
| **548** | 0.044 | 0.015 | 0.01 | 0.04 | 0.042 | 0.031 |
| **549** | 0.043 | 0.015 | 0.01 | 0.039 | 0.041 | 0.029 |
| **550** | 0.048 | 0.017 | 0.011 | 0.044 | 0.046 | 0.033 |
| **556** | 0.053 | 0.018 | 0.013 | 0.049 | 0.05 | 0.037 |
| **558** | 0.051 | 0.017 | 0.011 | 0.046 | 0.049 | 0.035 |
| **560** | 0.047 | 0.016 | 0.011 | 0.044 | 0.047 | 0.034 |
| **561** | 0.051 | 0.017 | 0.012 | 0.047 | 0.049 | 0.035 |
| **586** | 0.037 | 0.013 | 0.01 | 0.032 | 0.033 | 0.025 |
| **602** | 0.026 | 0.011 | 0.008 | 0.024 | 0.027 | 0.02 |
| **603** | 0.033 | 0.014 | 0.011 | 0.03 | 0.033 | 0.025 |
| **612** | 0.036 | 0.013 | 0.01 | 0.031 | 0.034 | 0.025 |
| **644** | 0.086 | 0.024 | 0.017 | 0.069 | 0.07 | 0.05 |
| **650** | 0.081 | 0.023 | 0.016 | 0.066 | 0.067 | 0.047 |
| **662** | 0.095 | 0.026 | 0.019 | 0.077 | 0.076 | 0.055 |
| **673** | 0.062 | 0.02 | 0.015 | 0.052 | 0.051 | 0.039 |
